# Aberrant Uteroplacental and Vascular Signaling and Remodeling by Matrix Metalloproteinases in Pregnancy-Related Hypertension and Preeclampsia

**DOI:** 10.3390/biom16030380

**Published:** 2026-03-03

**Authors:** Ellie Y. Wu, Raouf A. Khalil

**Affiliations:** Vascular Surgery Research Laboratories, Division of Vascular and Endovascular Surgery, Brigham and Women’s Hospital, Harvard Medical School, Boston, MA 02115, USA; ellieywu2@gmail.com

**Keywords:** blood vessels, contraction, cytokines, estrogen, growth factors, hypertension, hypoxia, placental ischemia, progesterone, uterus

## Abstract

Normal pregnancy is associated with uterine and vascular remodeling by matrix metalloproteinases (MMPs) to facilitate placental blood flow and uterine expansion for the growing fetus. Increases in MMP-2 and MMP-9 in response to estrogen and progesterone promote placentation, uteroplacental vascularization and fetal growth during healthy pregnancy, but are altered in preeclampsia (PE). PE is characterized by hypertension in pregnancy (HTN-Preg) and fetal growth restriction (FGR). Predisposing genetic, demographic and environmental factors alter uteroplacental MMPs, immune response and integrins leading to apoptosis of invasive trophoblasts, inadequate spiral arteries remodeling, and reduced uteroplacental perfusion pressure (RUPP). Ensuing placental ischemia causes imbalance between anti-angiogenic soluble fms-like tyrosine kinase-1 (sFlt-1) and pro-angiogenic placental growth factor (PlGF) and promotes the release of tumor necrosis factor-α (TNF-α), hypoxia-inducible factor, reactive oxygen species, and angiotensin AT_1_ receptor agonistic autoantibodies. Systemically, these bioactive factors target vascular endothelial cells, smooth muscle cells, and extracellular matrix, causing endothelial dysfunction, vasoconstriction, inadequate vascular remodeling, and HTN-Preg, while locally they diminish uteroplacental remodeling and cause FGR. In support, animal models of HTN-Preg induced by RUPP or infusion of sFlt-1 or TNF-α show decreases in vascular MMP-2, MMP-9 and vasodilation, increases in MMP-1, MMP-7 and vasoconstriction, collagen accumulation, and arterial stiffness. Also, decreases in uterine MMP-2 and MMP-9 could impede uterine expansion and lead to preterm birth. Conversely, PlGF and TNF-α antagonist reversed MMPs imbalance and collagen accumulation, and improved vascular function, blood pressure, and pup weight in HTN-Preg models. Persistent postpartum changes in MMPs could affect maternal hemorrhage, future pregnancies, and HTN, and cause fetal programming of cardiovascular and metabolic diseases. Understanding the aberrant uteroplacental and vascular signaling and remodeling by MMPs could help design new biomarkers and remedies for PE. Targeting bioactive factors and rectifying MMP imbalance could improve vascular and uteroplacental remodeling, and manage HTN-Preg, FGR and PE.

## 1. Introduction

Normal pregnancy (Norm-Preg) is associated with multiple uteroplacental and hemodynamic changes to meet the metabolic demands of the growing fetus. The Preg uterus undergoes enlargement and expansion to provide enough space for fetal growth. Placental remodeling and trophoblast invasion of spiral arteries maintain sufficient blood supply for fetal development [1,2]. Pregnancy-related increases in the renin-angiotensin system and salt and water retention increase maternal blood volume and cardiac output, which are counterbalanced by estrogen (E2)-mediated systemic vasodilation and decreased vascular resistance, leading to a slight decrease in blood pressure (BP) mainly during mid-gestation [3,4]. These pregnancy-related changes in the uterus, placenta, and the vasculature involve significant uteroplacental and vascular remodeling by metabolic and proteolytic enzymes in different maternal tissues [5,6].

Matrix metalloproteinases (MMPs) are a family of zinc-dependent proteases that play a major role in extracellular matrix (ECM) turnover and tissue remodeling [7,8]. MMPs include collagenases, gelatinases, stromelysins, matrilysins, and membrane-type MMPs (MT-MMPs), with variable tissue distribution, mRNA expression, protein levels, and specific substrates [9]. Most MMPs are produced as inactive proMMPs that are cleaved by other proteases or MMPs into active MMPs [9,10]. Activated MMPs proteolyze different ECM protein substrates such as collagen and elastin [9]. MMPs play important roles in the female reproductive system, including remodeling of the endometrium during the menstrual cycle and estrous cycle, and remodeling of the uteroplacental vasculature and the myometrium to maintain healthy pregnancy [8].

In 5 to 8% of pregnancies, women present with HTN-Preg in one of four forms: chronic HTN that predates pregnancy, preeclampsia (PE)-eclampsia, chronic HTN with superimposed PE, and nonproteinuric gestational HTN [11]. PE is manifested as new onset HTN-Preg (systolic BP ≥ 140 mmHg and/or diastolic BP ≥ 90 mmHg) at ≥20 weeks of gestation, frequently near term, with or without proteinuria, and occasionally edema and increased platelet aggregation [12]. PE may also be associated with hemolysis elevated liver enzymes low platelets (HELLP) syndrome. PE could progress to eclampsia with severe HTN, seizures, coma, and death (approximately 14% of pregnancy-related maternal mortality) [13].

Many cases of PE are associated with FGR, preterm birth, small for gestational age birth weight, and neonatal death [11,14]. Preterm birth occurs in 10% to 15% of all pregnancies and is a major cause of perinatal morbidity and death [15]. A study of spontaneous births showed that 3.2% of preterm births and 2.2% of extreme preterm births are related to PE [16]. Perinatal complications are compounded in underdeveloped countries, which have higher incidence rates of PE, maternal mortality and preterm births than developed countries [17]. PE could also cause in utero fetal programming of cardiovascular and metabolic disease, and predisposition of the offspring to HTN and diabetes when they reach adulthood [18,19].

Although PE represents a major cause of maternal and fetal morbidity/mortality and imposes a large burden on the healthcare system, its etiology and underlying pathophysiology are not clearly understood. Importantly, PE-related increase in BP is reversed upon delivery of the baby and placenta, implicating the placenta and placental factors as central culprits in the disorder.

Mechanistic studies are essential to understand the underlying mechanisms of PE, but they are extremely difficult to perform on Preg women and therefore have been largely conducted in animal models of HTN-Preg. The current knowledge suggests that predisposing genetic and hereditary background reinforced by environmental risk factors initiate a localized reduction in uteroplacental perfusion pressure (RUPP) with ensuing placental ischemia/hypoxia [20,21]. Experimental studies support that placental ischemia is an initiating pathogenic event of PE [22,23,24]. The RUPP model of placental ischemia induced by clipping the lower abdominal aorta and the uterine branches of the ovarian arteries in late Preg sheep, dog, rabbit and rat shows some of the hallmarks of PE including HTN-Preg and FGR [20,22,23,24,25]. Specifically, BP is increased, and the litter size and individual pup weight are reduced in RUPP versus Norm-Preg rats [8,26].

**Figure 1:**
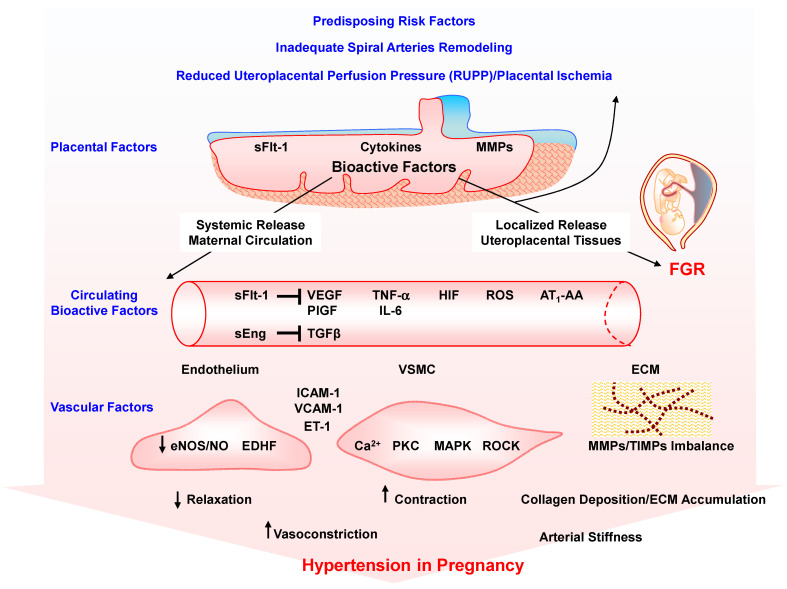
Pathophysiological mechanisms of HTN-Preg and FGR. Predisposing genetic, immune, demographic and environmental factors cause initial RUPP and placental ischemia, triggering the release of bioactive factors into the systemic circulation where they target the endothelium causing EC dysfunction, decreased endothelium-dependent vascular relaxation pathways, increased ICAM-1 and VCAM-1 adhesion molecules, endothelin-1 (ET-1) and mechanisms of VSMC contraction, and ECM causing MMPs/TIMPs imbalance and collagen deposition, and resulting in increased vasoconstriction, arterial stiffness and HTN-Preg. Locally, increases in bioactive factors target MMPs in the uteroplacental interface further diminishing uteroplacental remodeling and exacerbating placental ischemia and FGR. EDHF, endothelium-derived hyperpolarizing factor; PKC, protein kinase C; ROCK, Rho-kinase.

Studies in PE women and RUPP HTN-Preg rats also show imbalance in the circulating levels of pro-angiogenic vascular endothelial growth factor (VEGF) and PlGF and the anti-angiogenic factors sFlt-1 and soluble endoglin (sEng), and increases in the pro-inflammatory cytokines TNF-α and interleukin-6 (IL-6), hypoxia-inducible factor (HIF), reactive oxygen species (ROS) and angiotensin II (Ang II) type 1 receptor (AT_1_R) agonistic autoantibodies (AT_1_-AA) [25,27,28,29,30,31,32,33,34] (Figure 1). The release of these bioactive factors into the circulation targets various systemic vessels causing generalized vascular dysfunction and HTN-Preg, or the renal vessels and glomeruli causing glomerular endotheliosis, increased glomerular permeability and proteinuria, or the cerebral vasculature causing cerebral edema and seizures [11,14,21]. Also, the release of the bioactive factors locally causes further RUPP and exacerbates placental ischemia and FGR, leading to preterm birth and small for gestational age birth weight.

Because MMPs promote uteroplacental and vascular remodeling, healthy pregnancy and normal fetal growth, changes in MMP expression/activity could adversely affect vascular and uterine function and remodeling, thus contributing to the pathogenesis of PE. This prompted research to measure the levels of MMPs and examine the effects of sex hormones and growth factors on MMPs, and uteroplacental and vascular remodeling during healthy pregnancy. Additional research highlighted potential predisposing risk factors triggering changes in uteroplacental and vascular MMPs and placental ischemia in the settings of HTN-Preg, FGR and PE, and examined the effects of bioactive factors systemically on vascular ECs, VSMCs, ECM, and collagen, and locally on the myometrium and uterine contraction mechanisms, as well as the immediate postpartum and long-term effects on maternal and offspring health.

In this review, we discuss reports published in the PubMed database and data from our laboratory to highlight the changes in MMP expression/activity in the blood vessels, uterus and placenta during Norm-Preg and PE, and how predisposing risk factors, circulating bioactive factors, aberrant uteroplacental and vascular signaling mechanisms, and abnormal uteroplacental and vascular remodeling by MMPs could lead to HTN-Preg and FGR. As a narrative review, our goal was to synthesize key perspectives rather than exhaustively include all the literature. The review cites 568 articles, selected following three steps: (1) Database search: The PubMed database was searched for relevant articles published between 2000 and 2025 using the primary keywords pregnancy and preeclampsia. This initial research yielded 40,649 articles. (2) Screening: Careful screening excluded non-English articles, case reports, and small sample size research without justification. When more than one article addressed the same topic, the more recent article was selected. If an article referred to an original seminal discovery from earlier years, the original article was reviewed and included in the references. This narrowed the search to 2000 articles for further review. (3) Final inclusion: 568 articles were cited based on relevance, impact, expert input and novelty. The cited articles represent the most impactful and evidence-supported research work in the field. We will first describe the MMP levels during Norm-Preg, and in PE women and animal models of HTN-Preg. We will discuss how genetic, immune, demographic and environmental risk factors could alter uteroplacental MMPs, the immune response, and integrins and leading to apoptosis of invasive trophoblasts, deficient placentation, inadequate spiral arteries remodeling, and placental ischemia. We will follow with describing the release of various bioactive factors that target vascular ECs, VSMCs and MMPs and alter vascular function, ECM turnover and remodeling, leading to HTN-Preg. We will also describe how localized changes in uterine MMPs could affect uteroplacental remodeling and uterine contraction leading to FGR and preterm birth. We will then discuss the postpartum changes in PE women, the short-term and long-term maternal complications, and in utero fetal programming of cardiovascular and metabolic disorders in the offspring. We will also highlight the current approaches to managing PE and how understanding the role of bioactive factors and vascular and uteroplacental MMPs could help design new biomarkers for the diagnosis and novel approaches for the management of PE.

## 2. Vascular and Placental Changes in Association with MMPs During Norm-Preg

Norm-Preg is associated with marked vascular and uteroplacental changes to ensure embryo implantation in the uterine wall, adequate placentation, and sufficient nutrients and blood supply to the developing fetus. Preg-related increases in the female sex hormones E2 and progesterone (P4) inhibit vascular contraction mechanisms, promote vasodilation of the maternal uterine, renal and systemic vessels [35,36,37], and induce relaxation of rat aorta and uterine artery VSMCs [38,39]. Also, during Norm-Preg, extravillous trophoblasts (EVTs) invade the maternal decidua and spiral arteries, replacing ECs and VSMCs, thus creating large, dilated vessels, and ensuring adequate placentation and maintained nutrient supply to the fetus. Various proteolytic enzymes including MMPs degrade ECM proteins and facilitate trophoblast invasion into the decidual stroma. For instance, MMP-2 (gelatinase A) and MMP-9 (gelatinase B) promote endometrial tissue remodeling during the estrous cycle, menstrual cycle, and healthy pregnancy [40,41,42]. In support, MMP-2 and MMP-9 are abundantly expressed in invading EVTs and enhance their invasiveness capacity [43,44,45,46]. MMP-2 expression is also abundant in the umbilical cord [11], and serum levels of MMP-9 are elevated in Norm-Preg women [7]. The pregnancy-associated increases in E2 and P4 could influence the expression/activity of vascular MMPs. E2 enhances MMP-2 release from human umbilical artery VSMCs [47]. MMP-2 and MMP-9 levels were also increased in the aorta of Preg rats, and treatment of the aorta of non-Preg rats with E2 + P4 enhanced MMP-2 and MMP-9 activity [48]. Other factors such as epidermal growth factor (EGF) promote trophoblast invasion likely by increasing the expression/activity of MMP-2 and MMP-9 [49,50]. The increases in vascular MMPs largely degrade ECM proteins and promote vascular remodeling, angiogenesis, and the systemic vascular changes during pregnancy [51]. In addition, MMPs may affect vascular protease-activated receptors and other signaling mechanisms [52,53,54]. These observations support a role of MMPs in promoting systemic vasodilation during pregnancy.

Pregnancy-associated changes in MMPs could involve extracellular MMP inducer (EMMPRIN, CD147), a membrane protein of the immunoglobulin superfamily widely expressed in various tissues, including ECs, which affects MMP levels and tissue remodeling in pathological conditions such as atherosclerosis, heart failure, rheumatoid arthritis, and cancer [55,56,57,58,59,60]. EMMPRIN induces the release of MMP-1, MMP-2, MMP-3, and MMP-9 [61]. Our research showed increases in EMMPRIN, MMP-2 and MMP-9 levels in the aorta of late-Preg rats and virgin rat aorta treated with E2 + P4, which were blocked by an EMMPRIN neutralizing antibody, supporting a role of EMMPRIN in mediating the increases in vascular MMP-2 and MMP-9 levels during pregnancy and in response to E2 and P4 [48].

Studies also suggest a role of MMPs in placental remodeling during pregnancy [62]. Serum MMP-2 and MMP-9 levels are elevated in Preg compared to non-Preg bitches [63]. Also, MMP-2, MMP-14 and EMMPRIN expression is augmented in late-Preg bovine placenta [64]. Other studies showed increases in MMP-2 and MMP-9 in the placenta of diabetic mid-Preg rats [62] but did not track MMP changes in late-pregnancy. Interestingly, our research showed a decrease in placental MMP-2 and MMP-9 in late compared to mid-Preg rats [48]. The differences in placental MMP expression could be related to species differences in MMP regulatory mechanisms at different stages of pregnancy. Importantly, placental remodeling mainly occurs during embryo implantation and organ development in early and mid-gestation. In support, we observed parallel decreases in placental levels of MMP inducer EMMPRIN and MMPs in late-Preg compared to mid-Preg rats [48]. Collectively, these observations support a role of MMP-2 and MMP-9 in mediating vascular and placental remodeling during Norm-Preg, but do not minimize the involvement of other MMP subtypes.

## 3. Circulating, Vascular, and Uteroplacental MMPs in HTN-Preg and PE

The extensive uteroplacental and vascular remodeling by MMPs during Norm-Preg have suggested potential changes in MMPs that cause inadequate tissue remodeling in PE. Studies have measured serum and plasma MMP levels in PE compared to Norm-Preg women, but the results have been inconsistent. Some studies showed higher serum/plasma levels of MMP-2 in PE versus Norm-Preg women particularly during 2nd and 3rd trimesters [65,66,67] (Table 1). Other studies showed lower serum/plasma MMP-9 levels in PE compared to Norm-Preg women [65,68]. The causes of the discrepancies in MMP levels may be related to assay variability, gestational age stratification, PE subtypes, and maternal comorbidities. Importantly, the discrepancies could be due to the timing of measurement of MMP levels as they continuously fluctuate throughout pregnancy. For instance, one study found that serum MMP-9 levels were higher at the onset of PE but were lower in the 2nd and 3rd trimesters when compared to Norm-Preg women [67]. Another study showed decreased circulating levels of MMP-9 in early onset-PE (EO-PE) compared to late onset-PE (LO-PE), supporting the notion that MMP-9 is heavily involved in spiral artery remodeling in early pregnancy [65]. Other studies showed higher serum MMP-3 levels in EO-PE but not LO-PE, and elevated serum MMP-13 levels in both EO-PE and LO-PE compared to Norm-Preg women [68]. Further studies are needed to confirm MMP-3 and MMP-13 levels as they are not as commonly measured as MMP-2 and MMP-9 when investigating PE.

Discrepancies may also be caused by differences in MMP measurement techniques and potential interference of exogenous MMP inhibitors or endogenous tissue inhibitors of metalloproteases (TIMPs) with MMP activity. Importantly, increases in TIMPs could reduce MMP activity, and thereby affect ECM remodeling, arterial stiffness and vascular compliance in PE. Increased circulating TIMP-3 levels were observed in PE compared to Norm-Preg women with a positive correlation with plasma MMP-2 and TIMP-1 in PE [74]. DNA hypomethylation of the promoter region of placental *TIMP-3* gene increases TIMP-3 expression in PE [75]. Also, plasma/serum MMP levels represent the whole-body production of MMPs, and not their specific tissue levels. Studies showed lower expression of MMP-2, MMP-9, and urokinase-type plasminogen activator (uPA) and higher expression of TIMP-1, TIMP-2 and plasminogen activator inhibitor-1 (PAI-1) in postpartum placentae of PE compared to term Norm-Preg women [76]. Other studies showed decreased MMP-2, MMP-8, MMP-9, and MMP-11 and increased TIMP-1 and TIMP-3 in placental villous tissues of PE and FGR cases compared to Norm-Preg [77]. MMP-9 levels are also reduced in human trophoblasts from PE versus normal placentae [78]. Also, measurement of circulating and postpartum placental MMPs may not reflect their levels and roles in critical tissues at different stages of pregnancy. Animal models have been useful in determining the specific changes in MMPs in the uterus, placenta and blood vessels during HTN-Preg. Using gelatin zymography, Western blots and immunohistochemistry, we found that MMP-1 and MMP-7 levels were increased in the uterus, placenta, and aorta of RUPP compared to Norm-Preg rats (Table 2). The increase in MMP-1 and MMP-7 was associated with increased levels of collagen type I which could limit trophoblast invasion into the decidual stroma [79]. Additionally, the levels of proMMP-2, MMP-2 proMMP-9, and MMP-9 in the placenta and those of MMP-2 and MMP-9 in the uterus increased in the distal region compared to the proximal region of Norm-Preg rat uterus, suggesting a role in promoting vascular remodeling, placental development and vasculogenesis [80]. On the other hand, uterine, placental and aortic levels of proMMP-2, MMP-2, proMMP-9, and MMP-9 were consistently reduced in RUPP compared to Norm-Preg rats [8,80]. More thorough measurement of MMPs in different animal models of HTN-Preg could provide insights into the time course of the changes in MMPs in specific tissues during PE. However, animal models have their limitations as they do not accurately reflect all stages of human gestation due to species differences in hormonal and immunological status [81].

## 4. Predisposing and Risk Factors in PE and Their Link to MMPs

**Figure 2:**
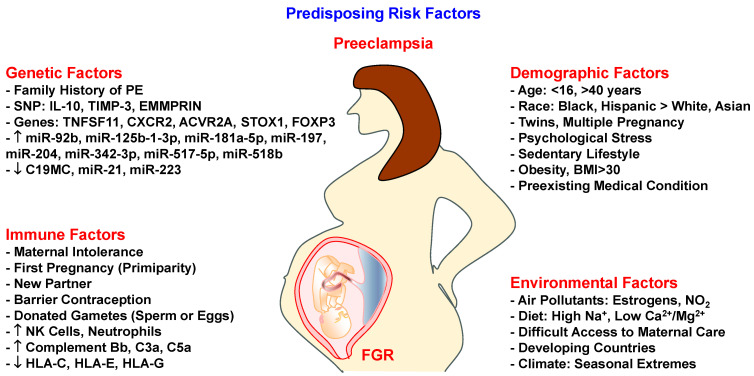
Risk factors of PE. Different genetic, immune, demographic, and environmental factors interact to increase a woman’s risk of developing PE. SNP, single nucleotide polymorphism; IL-10, interleukin-10; AVCR2, activin receptor II; STOX1, storkhead box 1; C19MC, chromosome 19 miRNA cluster; TNFSF11, tumor necrosis factor superfamily member 11; CXCR2, C-X-C chemokine receptor II; FOXP3, forkhead box P3.

The changes in MMP expression/activity in HTN-Preg and PE raise important questions regarding the predisposing risk factors that prompt these changes. Several genetic, immune, demographic and environmental factors could affect placental development and predispose Preg women to PE (Figure 2).

Family history of PE is an important predisposing hereditary factor. Females born from a PE-complicated pregnancy have a higher risk of developing PE in their pregnancies, suggesting inherited maternal genes [82]. Also, altered global gene expression in first-trimester placentae suggests that several placental genes could be mutated in PE [83]. Mutations in placental mitochondrial genes could disrupt the mitochondrial oxidation/reduction mechanisms leading to oxidative stress in the uteroplacental interface [84]. Also, several MMP genes are located in chromosome 11, indicating the sensitivity of this chromosome and consequently MMP expression to epigenetic changes and oxidative stress during PE [85]. Key inflammation-associated genes such as *INHBA*, *OPRK1*, *TPBG* are differentially expressed and contribute to the increased inflammatory response in PE [86]. Also, the tumor necrosis factor superfamily member 11 (*TNFSF11*) gene is linked to cytokine release from white blood cells with SNPs rs2200287 and rs2148072 associated with changes in the immune response and PE susceptibility [87]. Also, the anti-inflammatory cytokine interleukin-10 (IL-10) gene promoter –592A/C with the CC and AC + CC genotypes has been associated with elevated risk for PE [88]. Among Tunisian women, the L-10 −819 T/T variant and the ATA haplotype are associated with decreased production of IL-10 [89]. In addition, the C-X-C chemokine receptor type 2 (*CXCR2*) gene plays a role in inflammation and immunity, and SNP rs1126579 has been associated with increased PE risk [90,91,92]. Other PE susceptibility genes include activin receptor type-2A (*ACVR2A*) gene on chromosome 2q22 as part of the transforming growth factor-β (TGF-β) superfamily, and storkhead box 1 (*STOX1*) gene on chromosome 10q22, which encodes STOX1 DNA binding protein. *STOX1* Y153H polymorphism is linked to deficient trophoblast invasion and FGR, and is found in family trees encompassing several generations of women with EO-PE and severe PE [93]. In support, wild-type (WT) female mice bred with transgenic male mice overexpressing human *STOX1* show HTN-Preg and proteinuria [94]. Polymorphisms in *TIMP* genes could also influence the susceptibility to PE. For instance, TIMP-1 rs4898 C allele was associated with increased risk of EO-PE in a cohort of Preg Polish women [95]. Also, hypomethylation of TIMP-3 promoter in the placenta of PE women is linked to decreased VEGF binding to VEGFR and reduced angiogenesis, suggesting an epigenetic alteration underlying decreased trophoblastic invasion [96,97]. In support of epigenetic mechanisms, dysregulation of ten-eleven translocation 2 (TET2) is linked to hypermethylation of the MMP-9 promoter with consequent shallow trophoblast invasion in PE [98]. An rs424243T/G variant of the MMP inducer *EMMPRIN* gene is also overexpressed among PE women (55.6%) compared to normotensive women (16.7%) [69].

Research into microRNAs (miRs) supports a role of genetic factors in PE susceptibility and pathogenesis. Thirteen miRs (miR-92b, miR-197, miR-342-3p, miR-296-5p, miR-26b, miR-25, miR-296-3p, miR-26a, miR-198, miR-202, miR-191, miR-95, and miR-204) were overexpressed while miR-21 and miR-223 were underexpressed in PE compared with Norm-Preg women [99]. Dysregulation of placental chromosome 19 microRNA cluster C19MC impacts trophoblast differentiation, invasion, and angiogenesis [100]. Hypoxia downregulates C19MC expression causing inhibition of epithelial-to-mesenchymal transition genes, and subsequent decrease in EVTs migration and invasiveness [101]. Aberrant C19MC-derived miRs released into the maternal circulation in exosomes may target immune cells and other recipient cells where the transferred miRs interact with the cells’ mRNAs and alter their immune-specific functions [102]. In PE, increased placental expression of miR-125b-1-3p causes downregulation of sphingosine-1-phosphate receptor 1 (S1PR1), a G-protein-coupled receptor known to facilitate trophoblast invasiveness [103]. PE placentae also show upregulation of miR-518b which contributes to excessive trophoblast proliferation, while upregulation of miR-517-5p may decrease the trophoblast proliferative and invasive abilities [104]. The expression of miR-517a/b and miR-517c is also increased in placentae from PE vs Norm-Preg women, and in first-trimester primary EVTs exposed to hypoxia, resulting in decreased trophoblast invasion capacity [105]. Importantly, *FOXP3* gene plays a role in regulatory T cell (Treg) activation, and its downregulation/polymorphism could alter the maternal immune response, leading to decreased maternal tolerance and predisposition to PE [106,107]. *FOXP3* expression has been linked to increased MMP-2 and MMP-9 levels and cancer cell invasiveness [108], and downregulation of *FOXP3* and decreased MMP levels could contribute to diminished trophoblast invasiveness in PE. Other studies showed that MMP-9 was weakly expressed and negatively correlated with highly expressed miR-181a-5p in serum/placenta of women with severe PE compared with Norm-Preg women [109]. Also, small non-coding RNAs (sncRNAs) such as piwi-interacting RNAs (piRNAs) are upregulated in PE placentae, where they promote trophoblast apoptosis and inhibit their proliferation and invasion [110]. In support of immune factors, PE is more common in first pregnancy (primiparity), after a switch of partners, barrier contraception, and donated gametes, likely due to the semi-allogenic nature of the fetus or exposure to foreign material [11]. With regard to paternal genes, some studies showed a 2.7% PE risk in women with male partners whose mothers were diagnosed with PE compared with mothers who had a healthy pregnancy [111], but other studies suggest no effect of parental genes in the risk for EO-PE and only weak association with the risk for intermediate- and LO-PE [112]. Further studies of gene polymorphisms that regulate immunity, maternal tolerance and inflammation would help identify the specific genetic alterations that increase the risk for PE.

Maternal race/ethnic background, age, lifestyle, body weight, and multiple pregnancy are considered risk factors for PE [6]. The incidence of PE is greater in Preg African American (2.9%) than Asian women (1.2%) [113]. PE also more commonly occurs in Preg women < 16 or >40 years old. Studies in Finland and India showed a higher risk of PE in older than young women [114,115]. The incidence of PE is also increased to 7% in overweight women (body mass index, BMI, 30–34.9) and to 13% in obese women (BMI 50) as compared to ~3% in women with normal body weight (BMI, 18.5–24.9) [116]. Studies have suggested a relationship between maternal obesity, inflammatory cytokines and dysregulation of uteroplacental MMPs in PE, FGR, and gestational diabetes [117,118]. Preexisting conditions such as heart disease, chronic pulmonary disease, diabetes, renal disorders, systemic lupus erythematosus, psychological stress, reproductive system surgery and history of antepartum hemorrhage also increase the risk for PE [6]. Most cardiovascular and pulmonary disorders involve changes in tissue remodeling by MMPs, which could adversely affect vascular and uteroplacental remodeling during pregnancy and lead to PE.

Environmental factors such as climate, air quality, diet, and access to healthcare may influence the risk for PE. Exposure to environmental pollutants, fine particulate matter, and nitrogen dioxide increases PE risk [119]. Environmental estrogens may mimic or antagonize the vascular effects of E2, thereby influencing angiogenesis and vascular remodeling, and causing abnormalities in ECs and VSMCs growth/function, leading to PE [120]. Maternal high dietary salt intake and low magnesium or calcium intake have also been associated with PE [121,122]. In terms of geographical distribution, the incidence of PE is greater in developing (1.8–16.7%) than developed countries (0.4%) likely due to inadequate or underutilized prenatal facilities due to distance and cost [123,124,125]. Among Eastern countries, the Philippines had the highest incidence of PE likely due to advanced maternal age and variants of the *VEGF-A* and *VEGFR1* genes [125,126]. For the Western world, PE incidence is highest in Scandinavian countries like Norway and Finland, possibly due to seasonal extremes in cold weather and short daylight hours, with subsequent decreases in exposure to ultraviolet rays, and potential deficiencies in vitamin D and calcium [125,127]. These predisposing factors contribute to the overall risk for PE through alterations in maternal tolerance, immunity, and tissue remodeling by MMPs, making it important to delve deeper into how changes in MMPs and the immune response could affect the initial gestational stages and the pregnancy outcomes.

## 5. Abnormal Placentation and Placental Ischemia in PE

**Figure 3:**
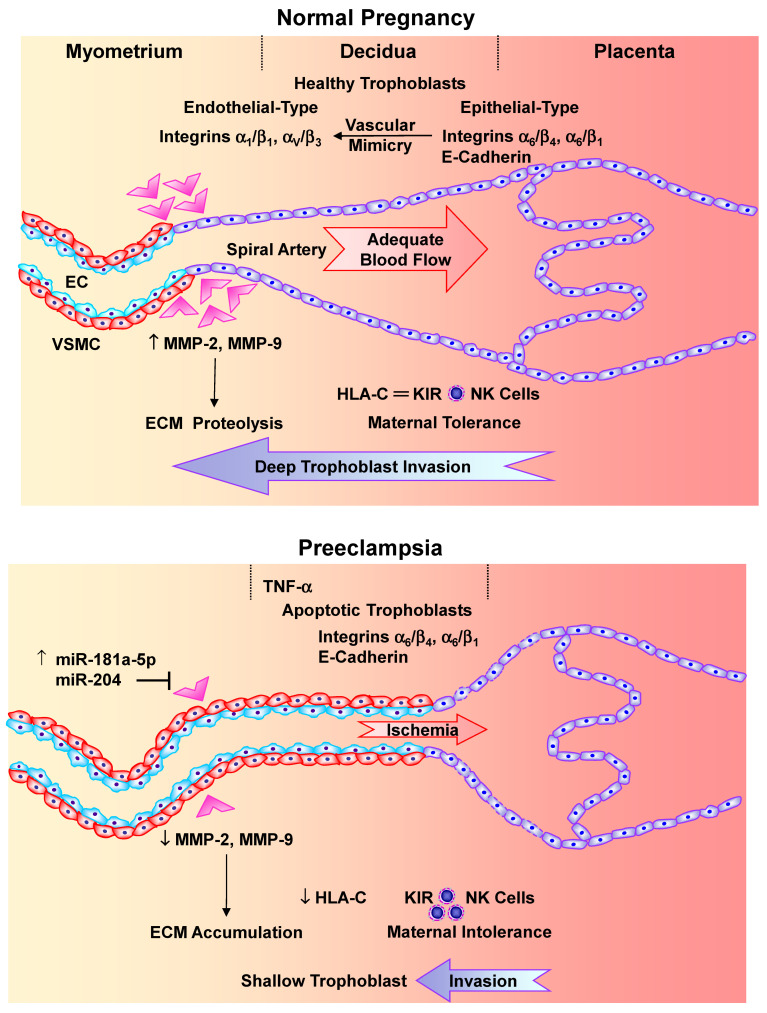
Deficient placentation in PE. During Norm-Preg, trophoblasts initially express the epithelial-type adhesion molecules integrins α_6_/β_4_ and α_6_/β_1_, and E-cadherin. As trophoblasts become invasive, they express endothelial-type integrins α_1_/β_1_ and α_V_/β_3_ (“vascular mimicry”). Also, increases in MMP-2 and MMP-9 enhance ECM proteolysis and facilitate vascular remodeling. Trophoblasts overexpress HLA-C that interacts with the inhibitory KIR receptor and decreases natural killer (NK) cells, thus contributing to maternal tolerance. As a result, trophoblasts invade the decidua to one-third of the myometrium, causing extensive remodeling of the spiral arteries from small-caliber resistance vessels to high-caliber capacitance vessels, thus providing adequate placental blood flow. In PE, increased miR-181a-5p and miR-204 decreased MMP-2 and MMP-9, leading to ECM accumulation that hinders vascular remodeling. Decreased HLA-C interaction with the inhibitory KIR receptor increases NK cells and decreases maternal tolerance. Increased immune response causes the release of TNF-α, apoptosis of trophoblasts and maintained expression of epithelial-type integrins α_6_/β_4_ and α_6_/β_1_, and E-cadherin. The decrease in trophoblast invasion of spiral arteries leads to shallow placentation to only superficial layers of the decidua, resulting in decreased blood flow and placental ischemia.

In the early stages of pregnancy, vasculogenesis, angiogenesis, trophoblast invasion and vascular remodeling contribute to the development of the placenta as an interface between the maternal and fetal circulations. Vasculogenesis entails the development of de novo vessels from pluripotent mesenchymal stem cells at 18–35 days following conception. Concomitantly, angiogenesis, sprouting and branching of preexisting uteroplacental vessels in response to pro-angiogenic growth factors and enhanced invasive abilities of trophoblasts promote placental vascularization and maintain healthy pregnancy [128]. During the first trimester of Norm-Preg, placental EVTs invade deeply into the maternal decidua up to one-third of the myometrium wall, progressively replacing ECs and VSMCs in the spiral arteries, and transforming the elastic tissue into fibrinoid material [129]. This results in progressive dilation and conversion of spiral arteries from high-resistance low-capacity to low-resistance high-capacity blood vessels, allowing sufficient blood and nutrient supply to the fetus (Figure 3).

Because of the increases in BP associated with PE remit following delivery of the baby and the placenta, the placenta is thought to be a major malefactor in the disorder. Transmission electron microscopy showed ultrastructural changes, distorted microvilli, frequent trophoblast cells with numerous vacuolated mitochondria lacking cristae, and few rough endoplasmic reticulum, lysosomes and glycogen deposits in PE compared to Norm-Preg placenta [130]. Inadequate placentation, RUPP and placental ischemia/hypoxia represent important events in the pathogenesis of PE [20,24,25]. Several factors can cause inadequate placentation including aberrant MMPs, abnormal immune responses, accumulation of natural killer (NK) cells and macrophages, trophoblasts apoptosis and decrease in their invasive capacity of the spiral arteries, and abnormal expression of integrins, leading to shallow trophoblast invasion of the decidua and myometrium and insufficient spiral arteries remodeling.

### 5.1. MMPs, Ovulation, Fertilization, Implantation, and Defective Placentation

Predisposing risk factors can adversely impact the female reproductive system even before placentation through alterations in ovulation, egg fertilization, and embryo implantation. During the menstrual/estrous cycle, ovulation is initiated by increases in luteinizing hormone which activates progestins, prostaglandins, and downstream MMPs leading to ECM remodeling, follicular layer rupture, and ovulation [131,132]. MMP-2 and MMP-9 are regulated by luteinizing hormone and progestins [133]. MMP-2-related type IV collagenolytic activity is found in follicular fluid, increases toward ovulation, and decreases rapidly as the follicles rupture, suggesting a role in follicular rupture and ovulation [134]. Higher levels of MMP-2, MMP-9, TIMP-1 and TIMP-2 were also detected in follicular fluid of women with polycystic ovary syndrome compared with control women, all undergoing in vitro fertilization (IVF) [135]. Increases in MMP-14 and MMP-16 levels in the ovarian tissue of naturally cycling women as well as macaque and rat models across the periovulatory period suggest a role of MT-MMPs in the ovulation process [136]. Changes in MMP expression/activity may alter the course of ovulation and subsequent conception. Egg fertilization by the sperm also involves MMPs. In Preg couples treated with intrauterine insemination, sperm MMP-2 and seminal plasma MMP-9 activity were higher in semen specimens from successful Preg compared to non-Preg group. There was also a correlation between sperm MMP-2 and MMP-9 activity and total antioxidant capacity of seminal plasma, supporting a role of MMPs in the outcome of pregnancy by intrauterine insemination [137]. Changes in MMP activity and egg fertilization may influence maternal tolerance toward the fetus and thereby contribute to the development of PE.

Following conception, endometrium-derived exosomes facilitate embryo implantation through crosstalk between the blastocyst and the endometrial epithelium. MMPs were detected in endometrium-derived exosomes, indicating a role in the blastocyst/endometrium epithelium crosstalk and embryo implantation [138]. Dysregulated MMPs are observed in recurrent implantation failure of IVF [139]. Also, epidemiological data suggest that IVF is associated with increased risk of placentation disorders including PE and FGR. For instance, superovulation could alter the expression of genes critical to endometrial remodeling such as MMPs during early implantation, leading to altered trophoblast migration and endovascular invasion and inadequate placentation [140]. Along with growth factors, cytokines and adhesion molecules, MMPs are major factors detected in the embryo implantation site [139], and dysregulation of MMPs can alter implantation and lead to defective placentation in PE.

MMP-2 and MMP-9 promote ECM remodeling and facilitate trophoblast invasion of the spiral arteries during Norm-Preg [43,44,45,46]. The levels of MMP-2 and MMP-9 are augmented in the aorta of Preg rats, suggesting their involvement in pregnancy-related vascular remodeling [48]. In contrast, the expression of bone marrow stromal cell antigen 2 (BST2) and MMP-2 is downregulated in placentae of PE versus Norm-Preg women. In HTR-8/SVneo and JAR cells, overexpression of BST2 upregulated MMP-2 expression and enhanced cell migration and invasion capacity. Downregulation of MMP-2 decreased the invasion capacity of HTR-8/SVneo cells, while MMP-2 overexpression reversed this effect [141]. Also, MMP-9 knockout (KO) mice show a PE-like phenotype possibly due to decreased trophoblast differentiation and invasion of spiral arteries [142]. Additionally, the invasive capabilities of BeWo trophoblast-like cells were suppressed by miR-204 mimics likely through downregulation of MMP-9 [143]. Taken together, these observations suggest a relationship between decreased MMP-2 and MMP-9 levels and inadequate trophoblast invasion of spiral arteries in PE.

Other MMPs are expressed in decidual and placental trophoblasts, altering their invasive capacity and uteroplacental remodeling in PE [144]. Some studies showed lower levels of the collagenase MMP-1 in the decidua, umbilical cord blood, and placenta of PE versus Norm-Preg women, which correlated with PE severity [71]. Also, the matrilysin MMP-7 is involved in endometrial tissue remodeling and rat estrous cycle, and its strong expression in the mature decidua of early Preg rats suggests its importance in decidual remodeling during pregnancy [145]. MMP-10 is also widely expressed in first-trimester decidual tissue including trophoblasts and ECs, but its expression is markedly lower in pregnancies at risk of PE compared to healthy pregnancies [146]. First-trimester trophoblasts from human placenta and cultured human aortic VSMCs release MMP-12, which could promote elastolysis and spiral artery remodeling during pregnancy [147]. Notably, serum MMP-12 levels decrease sharply from the first trimester to the second trimester in Norm-Preg women indicating a prominent role during early placentation and a declining role with successful placentation [148]. MMPs and their role in trophoblast invasion are regulated by miRs and other gene promoters. For instance, miR-675-5p-mediated GATA2 inhibition has been implicated in upregulating MMP-13 and MMP-14 and enhancing the invasiveness of EVTs isolated from first-trimester human placentae [149]. Also, bone morphogenetic protein 2 (BMP2) upregulates lncRNA NR026833.1 and promotes SNAIL expression to induce MMP-2 expression and facilitate EVT invasion, and BMP2 dysregulation may interfere with early placental development in EO-PE [150,151,152].

Interestingly, the uterus, placenta, and aortic tissue weight and histological cross-sectional area are reduced in RUPP compared to Norm-Preg rats. Also, MMP-2 and MMP-9 are abundantly expressed in uteroplacental and vascular tissues of Norm-Preg rats, particularly in the aortic media supporting a role of MMPs in pregnancy-related remodeling, which is also in agreement with reports that VSMCs are a major source of MMPs [48,153,154]. Conversely, MMP-2 and MMP-9 levels were reduced in the uterus, placenta and aorta of RUPP compared to Norm-Preg rats. Also, the trophoblast marker cytokeratin-7 showed less staining and invasion of spiral arteries into the decidua of RUPP versus Norm-Preg rats [155], supporting a link between reduced MMP levels, decreased uterine, placental, and aortic tissue weight and cross-sectional area, and growth-restrictive remodeling in rat models of HTN-Preg [8].

### 5.2. Abnormal Immune Responses and Inadequate Placentation in PE

While pregnancy is a normal biological process, the development of the fetus in the uterus constitutes a challenge to maternal tolerance and the immune system. In order to maintain healthy pregnancy, the maternal immune system accommodates and tolerates the semi-allogenic fetus, and at the same time makes adjustments to protect the fetus from excessive immune response that could cause fetal rejection [156]. In PE, the maternal immune and inflammatory responses are augmented causing an increase in the production of pro-inflammatory cytokines TNF-α and IL-6 [157]. Also, as a crucial component of the immune response, CD4^+^T cells from PE women showed increases in TNF-α and IL-17 levels, and caused increases in BP and renal cortical ET-1 mRNA expression when injected in nude athymic rats [157]. In support of exaggerated immune and inflammatory responses in PE, women who test positive for human immunodeficiency virus (HIV) often have suppressed immune system and show lower incidence rates of HTN-Preg and PE [158].

In healthy pregnancy, trophoblasts express and release large amounts of the major histocompatibility complex molecules, HLA-C, HLA-E and HLA-G, which bind to their respective receptors KIR, CD 94/NKGs and ILT-2 on NK cells, reducing their activity and preventing them from attacking normal fetal and placental tissues [159]. A decrease in HLA-C interaction with KIR on NK cells would increase their activity and allow them to attack fetal and placental tissues, hindering placental development and causing PE [160], Likewise, soluble human leukocyte antigen-G (sHLA-G) is an alternative splicing variant of HLA-G expressed by trophoblasts and immune cells and mitigates the maternal immune response against fetal cells, thereby facilitating EVTs invasion of spiral arteries, arterial remodeling and placental perfusion. Decreases in maternal sHLA-G levels lead to unchecked, excessively activated immune system and PE [104].

Norm-Preg is also associated with modest activation of the complement system, and exaggerated release of complement activation products such as Bb, C3a and C5a has been associated with PE [161]. Also, immunohistochemical analysis of microvessels from human subcutaneous fat showed more neutrophils adherent to ECs in vessels from women with PE than Norm-Preg women, which could promote vascular inflammation and EC dysfunction in PE [162]. Interestingly, in RUPP rat model of placental ischemia, suppression of the innate immune responses by inhibiting complement activation or depleting neutrophils decreases BP, supporting the role of complement activation products in HTN-Preg and PE [161,163].

Inflammatory cells produce MMPs. During Norm-Preg, decidual macrophage-derived MMP-3 contributes to ECM degradation and spiral artery remodeling [164]. Macrophages and NK cells from first-trimester decidua of Norm-Preg women also express MMP-2, MMP-7, MMP-9, MMP-11, MMP-16, and MMP-19 [165]. In non-contact cell co-culture model, the culture medium of PMA-activated THP-1 monocytes into macrophages increased the production of MMP-2 and MMP-9 and reduced TIMP-1 and TIMP-2 levels in co-cultured HEC-1A endometrium cells [166]. These observations suggest a link between macrophages, intrinsic immune and NK cells, and MMPs in regulating the immune response and the placentation process. Inflammatory cells expression/release of MMPs is augmented in various autoimmune and inflammatory disorders and increases in the immune and inflammatory response could alter uteroplacental and vascular MMPs, leading to inadequate uteroplacental and vascular remodeling in HTN-Preg and PE.

### 5.3. Integrins and Reduced Trophoblast Invasion of Spiral Arteries in PE

Integrins and other adhesion molecules play a role in trophoblast invasion and remodeling of the spiral arteries. Initially, trophoblasts express epithelial cell-type adhesion molecules including integrins α_6_/β_4_ and α_6_/β_1_, and E-cadherin. During Norm-Preg, trophoblasts become more invasive and promote vascular mimicry or pseudovasculogenesis, a process whereby epithelial cell-type adhesion molecules undergo phenotypic switch to EC-type integrins α_1_/β_1_ and α_V_/β_3_. However, in PE, trophoblasts expressing α_V_ integrins start to differentiate along the invasive pathway, but do not complete the process [167]. Initial placental hypoxia may hinder the phenotypic switch from epithelial to endothelial integrins and thereby contribute to the pathogenesis of PE. In early placenta and choriocarcinoma cell line BeWo, hypoxia increased the expression of integrin α_5_ and fibronectin and decreased the expression of integrin α_1_ [168]. Trophoblast apoptosis and maintained expression of epithelial cell-type adhesion molecules limit their invasion of the spiral arteries, further contributing to placental ischemia and PE [167,169,170]. Ezrin, an integrin that facilitates cell adhesion, organization and migration, is downregulated in syncytiotrophoblast microvesicles from PE women, thus contributing to reduced trophoblast invasiveness, shallow placentation and inadequate vascularization [171]. The decrease in the trophoblast capability to invade and replace the vascular cells in the spiral arteries also leads to retention of VSMCs, thus promoting vasoconstriction and further decreasing uteroplacental blood flow and worsening placental ischemia [172].

Other adhesion molecules such as intercellular adhesion molecule-1 (ICAM-1) and vascular cell adhesion molecule-1 (VCAM-1) are downregulated during healthy pregnancy, thus reducing adhesion of leukocytes and inflammatory cells to ECs and maintaining patent spiral arteries. PE is associated with increases in the plasma levels of ICAM-1, thus promoting leukocyte adhesion to ECs and restricting uteroplacental blood flow [173].

The question arises as of how localized changes in uteroplacental MMPs, immune response and integrins and subsequent decrease in trophoblast invasion of spiral arteries and placental ischemia could lead to the systemic vascular dysfunction and HTN-Preg associated with PE.

## 6. Circulating Bioactive Factors and Effects on MMPs in PE

Placental hypoxia/ischemia is thought to stimulate the release of multiple bioactive factors including antiangiogenic factors such as sFlt-1 and sEng, pro-inflammatory cytokines such as TNF-α and IL-6, HIF, ROS and AT_1_-AA [6,27,28,29,32,174,175,176]. These bioactive factors could be released locally and target uteroplacental MMPs causing further vasoconstriction of spiral arteries and exaggerated placental ischemia, or could be released systemically, causing generalized vasoconstriction, increased BP, HTN-Preg and PE [14,177].

### 6.1. Decreased Pro-Angiogenic and Increased Anti-Angiogenic Factors in PE

#### 6.1.1. Vascular Endothelial Growth Factor (VEGF)

The *VEGF* gene promotes the expression of growth factors VEGF-A, VEGF-B, VEGF-C, VEGF-D, and PlGF [177]. VEGF-A, VEGF-B and PlGF interact with tyrosine kinase receptor Flt-1 (VEGFR-1). VEGF-A also interacts with VEGFR-2 (Flk-1 or KDR) to enhance placental vascularization [177]. VEGF regulates EC growth, angiogenesis and vascular permeability [14,177]. In ECs, VEGF stimulates cytosolic free Ca^2+^ concentration ([Ca^2+^]_c_), Ca^2+^/calmodulin interaction, endothelial nitric oxide synthase (eNOS), and prostacyclin (PGI_2_) synthesis [178,179,180]. In human umbilical vein ECs (HUVECs), VEGF also enhances Ca^2+^-independent formation of NO by increasing Akt activity and inducing phosphorylation of eNOS Ser^1177^ [180].

Measurements of circulating VEGF varied depending on the assay method and whether total or free VEGF was measured. Some studies showed elevated circulating VEGF levels in PE [181,182,183]. Also, VEGF release was greater in placental villous explants from PE than Norm-Preg women [184]. It is thought that the increased BP and vasoconstriction in PE would augment vascular shear-stress and promote EC release of VEGF into the circulation [6]. Studies also found that excess VEGF activated Flt-1 and KDR receptors in JEG3 and HTR-8/SVneo cells and enhanced sFlt-1 release [185]. Other reports found no change or decreased serum VEGF levels in PE [186,187]. Plasma VEGF levels are also reduced, but placental VEGF production is greater in RUPP versus Norm-Preg rats [25,186,188]. The divergence in VEGF measurements could be due to the observation that PE is associated with increases in circulating sFlt-1 which can bind VEGF, resulting in higher total (bound and free) VEGF levels as assessed by radioimmunoassay or competitive enzyme immunoassay, but lower free VEGF levels as assessed by enzyme-linked immunosorbent assay (ELISA) in PE women and HTN-Preg models versus Norm-Preg [189].

Decreased VEGF may cause glomerular endotheliosis and proteinuria in PE. Glomerular podocytes constitutively synthesize VEGF to maintain EC integrity and fenestrae formation. Genetic deficiency of glomerular VEGF is associated with glomerular endotheliosis, diminished fenestrae, and disrupted kidney filtration apparatus [190]. Also, the use of VEGF antibodies such as bevacizumab in cancer clinical trials is associated with proteinuria and HTN [191,192]. Additionally, in adult mice, conditional deletion of *VEGF* gene in renal podocytes causes a renal pathology akin to PE including thrombotic microangiopathy and glomerular injury preceding HTN and proteinuria, linking deficient VEGF to the proteinuria and other renal manifestations in PE [192]. Also, intraperitoneal infusion of recombinant VEGF_121_ in RUPP rats reduced BP and improved renal glomerular filtration rate, renal plasma flow, and acetylcholine (ACh)-induced relaxation of the carotid artery, suggesting that pro-angiogenic factors could ameliorate the glomerular endotheliosis associated with PE [193]. Moreover, injection of non-viral L-tyrosine polyphosphate (LTP) nanoparticles containing the plasmid DNA for VEGFR2 in the uterine wall of RUPP rats improved pup and placenta weight, and reduced BP and uterine artery myogenic reactivity, supporting the benefits of promoting VEGF/VEGFR2 interaction in PE [194].

Many cell types including ECs secrete VEGF and TGF-β in an autocrine or paracrine fashion to promote angiogenesis. In ECM, MMPs cleave growth factor-binding proteins, thus releasing growth factors and affecting cell growth/proliferation. In addition to releasing ECM-bound growth factors, MMPs promote VEGF angiogenic effects through increasing proteolytic activity to detach pericytes from the vascular wall and facilitate angiogenesis, unmasking pro-angiogenic integrin binding sites in ECM, increasing pro-migratory ECM fragments, and cleaving EC-cell adhesions [51]. Growth factors, in turn, can regulate MMPs. In cultured human retinal pigment epithelial cells, exogenous MMP-9 increased the gene expression and secretion of VEGF, while cobalt chloride-induced chemical hypoxia upregulated MMP-2 and MMP-9 mRNA expression, and VEGF increased mRNA expression and secretion of MMP-9 [195]. In rat VSMCs, PlGF-BB upregulates MMP-2 expression, likely through activation of ROCK, extracellular signal-regulated kinases (ERK), and p38 mitogen-activated protein kinase (MAPK) [196]. Also, in carotid plaques VSMCs, EGF increases MMP-1 and MMP-9 mRNA expression and MMP-9 activity [197]. In HUVECs, VEGF promotes the expression of MMP-1, MMP-3, MMP-7, MMP-8, MMP-9, MMP-10, MMP-13, and MMP-19, likely through PI_3_K and MAPK pathways [198]. MMP-9 also regulates VEGF activation of VEGF-R/neuropilin-1 signaling pathways that normally contribute to trophoblast migration/invasion, but are downregulated in PE placenta and in trophoblasts exposed to hypoxia [199]. Thus, disruption of VEGF and MMPs interactions could affect uteroplacental and vascular remodeling in PE.

#### 6.1.2. Placental Growth Factor (PlGF)

PlGF is an important pro-angiogenic factor in Norm-Preg. While PlGF affinity for VEGFR-1 is only 1/10th of VEGF’s affinity, it reinforces VEGF actions and stimulates EC growth, placental vasculogenesis, and uterine artery vasodilation [14,200]. Plasma PlGF levels are low (~44 pg/mL) in non-Preg women, but markedly increase to ~40 times those of VEGF in Norm-Preg women [200], and progressively increase from ~353 pg/mL in gestational weeks 21 and 22 to ~574 pg/mL at 29 and 30 weeks [201]. In contrast, plasma/serum PlGF levels decrease in PE [181,202,203,204], with greater decreases detected in EO-PE than LO-PE among Haitian women [205]. In EO-PE, placental endoplasmic reticulum stress and apoptosis of decidual cells and trophoblasts suppress PlGF transcription/release [206]. Among the four alternatively spliced PlGF mRNA variants, PIGF 1–4, the predominant PIGF-1 isoform is markedly downregulated in PE [189]. Plasma PlGF levels are also reduced in RUPP rats and deoxycorticosterone acetate (DOCA)-salt (0.9% saline in the drinking water) rat model of HTN-Preg [25,207].

PlGF not only promotes EC growth, but also enhances vasodilation through activation of VEGFR-1, EDHF, and small conductance Ca^2+^-activated K^+^ channels (SK_Ca_) [208,209]. In mesenteric microvessels of Preg rats pretreated with N_ω_-nitro-L-arginine methyl ester (L-NAME) and indomethacin, a second application of PlGF caused greater vasodilation and reduction in VSMC [Ca^2+^]_c_ than the first PlGF exposure. Like VEGF, PlGF causes VEGFR-1 dimerization, such that the initial PlGF application would promote the formation of VEGFR-1 homodimers and coordination of their submembrane signaling, thereby enhancing the vasodilator response of repeated PlGF application [209]. On the other hand, a decrease in plasma PlGF levels would decrease vasodilation and increase BP in HTN-Preg and PE.

#### 6.1.3. Soluble Fms-like Tyrosine Kinase-1 (sFlt-1)

Angiogenesis is regulated by a balance between pro-angiogenic and anti-angiogenic factors. sFlt-1 (sVEGFR-1) is a major anti-angiogenic factor expressed as an alternatively spliced variant of VEGFR-1 that maintains the binding capacity of VEGFR-1 but lacks both the transmembrane and cytoplasmic domains and stays soluble in the circulation. Being in the circulation, sFlt-1 acts as a decoy receptor that binds plasma VEGF and PlGF and prevents them from binding to and stimulating membrane VEGFR-1. Additionally, sFlt-1 may form a heterodimer with the membrane VEGFR-1 and block its post-receptor signaling [210]. Plasma sFlt-1 levels are very low, ~0.15 ng/mL, in non-Preg women, but trophoblasts enhance its expression and increase its plasma levels to ~1.5 ng/mL in Norm-Preg women [14]. Plasma sFlt-1 levels remain relatively stable during Norm-Preg with some increase in the third trimester and after gestational week 36. PE is associated with marked imbalance between sFlt-1 and proangiogenic VEGF and PlGF [181,186,203,205,211,212,213,214,215,216]. The *sFlt-1* gene locus is on chromosome 13q12. Women with trisomy 13 have an extra copy of the *sFlt-1* gene causing increases in circulating sFlt-1 levels, decreases in PlGF levels and a higher risk of PE [217]. Elevated circulating sFlt-1 levels were observed in EO-PE and LO-PE [181,202,203,204,205,216]. sFlt-1 levels are also higher in placental villous explants from PE versus Norm-Preg women [184].

Placental ischemia/hypoxia is thought to promote the release of HIF-1 which binds to the promoter region of *flt-1* gene and increases sFlt-1 expression [184,186]. Also, in EVTs, overexpression of miR-517a/b and miR-517c upregulates TNF superfamily member 15 (TNFSF15), causing Flt-1 splicing, and increased sFlt-1 production [105]. Additionally, sFlt-1 e15a is a splice variant of sFlt-1 abundantly expressed and released by syncytiotrophoblasts, causing a 10-fold increase in its serum levels in PE compared to Norm-Preg women. Furthermore, sFlt-1 e15a binds VEGF and reduces EC migration and tube formation [218].

PE placentae also show imbalance between proangiogenic and anti-angiogenic factors with a 53% and 70% decrease in VEGF/sFlt-1 and PlGF/sFlt-1 ratio, respectively [184]. Circulating sFlt-1/PlGF ratio is also elevated in PE versus Norm-Preg women from the second trimester onwards, and in EO-PE versus LO-PE, thus serving as a potential predictor of PE onset [204,205]. Other studies showed little differences in circulating sFlt-1/PlGF ratio in PE versus Norm-Preg women [219], questioning its validity as a PE biomarker. Interestingly, circulating sFlt-1 levels and sFlt-1/PlGF ratio are elevated in twin compared to singleton pregnancies, likely due to the larger placental mass in twin pregnancies [220,221], further supporting the concept that the placenta is an important source of sFlt-1 and a major culprit in PE. Also, plasma apheresis to remove sFlt-1 from PE patient circulation decreased sFlt-1/PlGF ratio, improved BP and prolonged pregnancy [222], providing further support of sFlt-1’s role in PE.

Experimental studies showed increases in placental and plasma sFlt-1 levels and sFlt-1/PlGF ratio in RUPP versus Norm-Preg rats [25,188,223]. Other animal models of HTN-Preg showed increases or modest changes in circulating sFlt-1 levels [207,224,225,226,227,228]. Induction of human sFlt-1 in Preg mice increased serum sFlt-1 levels in dams and sFlt-1 mRNA levels in both the placentae and fetuses, and led to FGR of all fetuses at term [229]. Also, infusion of sFlt-1 or adenoviral overexpression of sFlt-1 in Preg rats decreased plasma VEGF, increased BP and proteinuria, and caused glomerular endotheliosis, occlusion of renal capillaries and focal fibrin deposition in glomerular cells [186,230,231,232,233]. Additionally, treatment of HUVECs with conditioned medium from placental villous explants of PE women decreased angiogenesis, while removing sFlt-1 or application of VEGF or an sFlt-1 antibody reversed the anti-angiogenic effects and restored EC angiogenesis [184].

In cultured murine trophoblast cells, VEGF increased the expression of sFlt-1 mRNA, but not full-length Flt-1 mRNA. Also, transgenic overexpression of VEGF specifically in Preg mouse endometrium induced placental sFlt-1 production and elevated sFlt-1 levels in maternal serum [234]. It is thought that during Norm-Preg VEGF-induced release of sFlt-1 at the maternal–fetal interface may represent a localized feedback regulatory mechanism to prevent excess VEGF from damaging placental or fetal tissues [234], and dysregulation of this protective VEGF-sFlt-1 feedback pathway may contribute to the pathophysiology of PE.

The marked increases in circulating sFlt-1 in PE are believed to induce generalized endotheliosis in systemic vessels causing HTN-Preg, in the renal glomeruli causing proteinuria, and in the cerebral vessels causing seizures [233]. sFlt-1 may also target and modulate uterine, placental and vascular MMPs. For instance, in HUVECs, the adipokine visfatin upregulated VEGF, VEGFR-2, MMP-2 and MMP-9 and downregulated TIMP-1 and TIMP-2, while inhibition of VEGFR-2 and VEGF by sFlt-1 downregulated visfatin-induced MMP induction [235]. Also, in mice with induced abdominal aortic aneurysm, sFlt-1 treatment reduced the aneurysm size and MMP-2 and MMP-9 activity [236]. Importantly, treatment of uterine, placental and aortic tissues from Norm-Preg rats with sFlt-1 caused decreases in the expression/activity of MMP-2 and MMP-9 that were reversed by VEGF. Also, VEGF treatment of uterine, placental and aortic segments from RUPP rats increased MMP-2 and MMP-9 to levels similar to those observed in Preg rats [8]. Additionally, infusion of sFlt-1 in Preg rats increased BP and reduced MMP-2 and MMP-9 in the uterus, placenta and aorta to levels similar to those in RUPP rats. Furthermore, infusion of PlGF reduced BP and reversed the decreases in MMP-2 and MMP-9 and the increases in MMP-1 and MMP-7 and collagen-I and -IV in the aorta, uterus, and placenta of RUPP and sFlt-1 infused HTN-Preg rats [237]. These findings are in agreement with reports that infusion of VEGF decreased BP in the RUPP rat model of HTN-Preg [193].

#### 6.1.4. Soluble Endoglin (sEng)

In ECs, TGF-β1 binds to TGF receptors and stimulates cell proliferation/migration [177]. Endoglin (Eng) is a co-receptor for TGF-β1 and TGF-β3 abundantly expressed in the surface membrane of ECs and syncytiotrophoblasts, where it promotes cell proliferation and angiogenesis [238]. *Eng* gene mutations have been linked to loss of capillaries, hereditary hemorrhagic telangiectasia, and arteriovenous malformations [239]. In contrast with membrane-bound Eng, sEng is an anti-angiogenic factor that could bind TGF-β1 and prevent it from interacting with its natural angiogenic receptors, thus reducing the protective effects of TGF-β1 on eNOS activation and vasodilation [177]. Hypoxia increases sEng expression in placental explants, supporting its release in response to placental ischemia/hypoxia in PE [240].

Serum sEng levels are barely detectable in non-Preg women, and very low in Norm-Preg women [241], but are elevated 3-, 5- and 10-fold in women with mild PE, severe PE and HELLP syndrome, respectively [241]. Some studies showed increases in serum sEng levels accompanied by increased sFlt-1/PlGF ratio in both EO-PE and LO-PE [202,242]. Other studies at gestational weeks 10–17 showed increases in serum sEng levels in women who developed EO-PE, but not LO-PE [219], and the increases in serum sEng levels were associated with decreases in serum MMP-14, particularly in severe PE [243].

Increases in serum and placental sEng levels and decreases in serum TGF-β levels were detected in RUPP rats [32], but not in the L-NAME-treated or DOCA-salt HTN-Preg rats [207,224]. One possibility is that sEng mainly functions in synergism with sFlt-1 to enhance the overall effect on vascular permeability, severe HTN, proteinuria, and FGR [241]. This is supported by reports that infusing Preg rats with both sEng and sFlt-1 shows characteristics similar to HELLP syndrome, and increased urinary protein/creatinine ratio and ET-1 secretion [244]. Increased serum sEng targets different components of the blood vessels. In HUVECs, recombinant sEng reduced EC tube formation to the same extent as sFlt-1 [241]. Interestingly, HUVECs secrete substantial amounts of sEng that are further increased by coexpression of Eng and MMP-14 and reduced by MMP inhibitors or MMP-14 short hairpin RNA, suggesting Eng shedding by membrane-type MMP-14, a mechanism that could regulate ECs’ angiogenic potential [245], and alter vascular remodeling in HTN-Preg and PE.

### 6.2. Cytokines, TNF-α, and Interleukins

Throughout gestation, pro-inflammatory cytokines increase modestly to maintain innate and adaptive immunity during the heightened metabolic demands [246]. In PE, placental ischemia promotes the release of TNF-α and interleukins (ILs) [11,14,156,247]. Higher levels of TNF-α, LIGHT (TNF superfamily member 14), interferon-γ (IFN-γ), IL-2, IL-6 and IL-8 were observed in the serum of PE versus Norm-Preg women [202,246,248,249,250,251,252]. Increases in serum levels of TNF-α, IL-6, and IL-35, and placental MMP-12 transcripts were also detected in preterm PE compared to preterm Norm-Preg women [253]. In human placental explants subjected to hypoxia/reoxygenation, higher TNF-α levels were detected in the medium, but not in the tissue homogenates [254]. Also, no differences in TNF-α or IL-6 levels were observed in PE versus Norm-Preg placentae [252,255], suggesting other non-placental sources. Monocytes and macrophages are the first cells activated to produce pro-inflammatory cytokines in nonspecific immune response [256] and in PE. In support, cultured monocytes released greater amounts of TNF-α and IL-6 when treated with plasma from PE than Norm-Preg women [256].

Experimental studies showed that the CD4^+^T cell production and plasma levels of TNF-α were greater in RUPP compared to Norm-Preg rats [174,175,247,257,258]. Also, infusion of TNF-α in late Preg mice, rats, and baboons was associated with HTN and proteinuria [227,228,259]. Similarly, infusion of LIGHT in Preg mice caused increases in BP, proteinuria, sFlt-1, and ET-1 [251]. TNF-α may function synergistically with IL-6 to elevate ET-1 levels and BP in RUPP rats [247]. TNF-α may also work in concert with sFlt-1 to create a combined pro-inflammatory and antiangiogenic state that exacerbates HTN-Preg and PE. For instance, HUVECs treated with TNF-α and sFlt-1 show increased expression of the adhesion molecules ICAM and VCAM and augmented release of markers of EC dysfunction including ET-1 and von Willebrand factor [180]. Interestingly, treating RUPP rats with the TNF-α decoy receptor etanercept causes reduction in BP. Also, HUVECs show reduced ET-1 production when exposed to serum from RUPP rats treated with the TNF-α blocker etanercept than nontreated RUPP rats [247].

TNF-α promotes inflammation and increases vascular permeability, lymphocyte activation, IL-6 and IL-8 release, and fibroblast proliferation. Also, TNF-α downregulates eNOS and inhibits mitochondrial biogenesis, causing mitochondrial dysfunction, augmented ROS production, and oxidative stress [260]. TNF-α may also change the expression of adhesion molecules in uteroplacental vessels [247] and the release of MMPs in PE [261]. We have shown that TNF-α treatment decreased MMP-2 and MMP-9 and increased MMP-1 and MMP-7 in the aorta, uterus and placenta of Preg rats. Also, infusing RUPP rats with the TNF-α antagonist etanercept reversed the decreases in vascular and uteroplacental MMP-2 and MMP-9 and the increases in MMP-1, MMP-7, suggesting a role of TNF-α in MMP imbalance in HTN-Preg [79,262].

RUPP rats also show increases in plasma IL-6 levels [257,258]. Also, infusing Preg rats with IL-6 is associated with increases in BP, proteinuria, and vascular contraction and reduced endothelium-dependent NO-cyclic guanosine monophosphate (cGMP)-mediated vascular relaxation [28,263]. IL-6 binds to IL-6R, and the IL-6/IL-6R complex associates with the glycoprotein gp130 in hematopoietic progenitor cells, ECs, and VSMCs, which dimerizes and initiates intracellular signaling and changes in vascular function [264]. IL-6 also causes endothelial barrier dysfunction and increases endothelial permeability through alterations in the ultrastructural distribution of tight junctions and changes in EC morphology [265].

IL-1β also contributes to the inflammatory response and disruption of EC function in PE. In support, IL-1β release is greater in monocytes from PE than Norm-Preg women [266]. Porcine endometrial tissues showed changes in MMP-2, MMP-9, MMP-12 and MMP-13 mRNA expression during the estrous cycle and pregnancy, and treatment of endometrial explants with IL-1β increased the expression of MMP-2, MMP-8, MMP-9, and MMP-13, while IFN-γ increased MMP-2 expression [267]. Also, IL-8 and MMP-2 were elevated in HUVECs from PE women than those from Norm-Preg women, and inhibiting IL-8 decreased MMP-2 levels in HUVECs from PE women, supporting an upstream inflammatory pathway that alters MMP expression [268]. MMPs’ proteolytic activity is thought to promote the release of activated cytokines in PE [30].

During Norm-Preg, the anti-inflammatory cytokine IL-10 regulates monocytes-induced inflammatory response and reduces TNF-α and IL-1β expression [269]. IL-10 levels are decreased in the plasma and placenta of PE women and RUPP rats [249,253,255,257]. Also, exposure of normal term placental trophoblasts to hypoxia increases the release of pro-inflammatory cytokines and decreases IL-10 [270]. Lower serum IL-10 levels in the second trimester may serve as an early predictor of PE [246]. Importantly, uric acid stimulates monocytes to release cytokines, with hyperuricemia correlating with PE severity [271], and monocytes from PE women release more TNF-α and IL-1β than monocytes from Norm-Preg women [266]. An imbalance between pro-inflammatory cytokines and immune regulatory factors such as IL-10 and Tregs is considered a key contributor to PE [272].

### 6.3. Hypoxia Inducible Factor (HIF)

HIF is a transcriptional factor that contributes to the physiological response to hypoxia. HIF-1 is a heterodimer comprising oxygen (O_2_)-sensitive subunits HIF-1α and HIF-2α and a constitutive subunit HIF-1β. HIF-1 directly or indirectly regulates more than 2% of human genes including VEGF, leptin, TGF-β3, and NOS in ECs and other cells [11]. In Preg mice, increases in E2 and P4 upregulate uterine HIF-2α and HIF-1α, respectively [273]. HIF expression and circulating HIF-1α levels are further upregulated in PE [274,275], where they contribute to PE by promoting sFlt-1, sEng, ET-1, Ang II-converting enzyme (ACE) and Ang II production, and reducing the invasion capability of trophoblasts [11,276].

Placental HIF-1α levels are increased in RUPP rats [32]. Also, trophoblast-specific HIF-1α expression in Preg mice led to FGR, decreased pup birth weight, reduced placental branching morphogenesis, altered maternal and fetal blood space, and reduced spiral arteries remodeling [277]. HIF-1α overexpression could activate several downstream pathways that lead to HTN-Preg and FGR. On the other hand, inhibiting placental HIF-1α expression using siRNA reduced BP, kidney damage, and serum sFlt-1 levels in HTN-Preg mouse model [278].

In addition to HIF regulation by O_2_, non-hypoxic stimuli including cytokines such as TNF-α could upregulate HIF-1α expression [228]. Also, hormones, metallic ions, and mechanical stimuli induce HIF expression [279,280]. In human placenta explants, treatment with LIGHT or AT_1_-AA induced HIF-1α in a hypoxia-independent manner [278]. Also, HIF-1α expression is increased in the internal spermatic vein of patients with varicocele [281]. HIF-1α and HIF-2α are also upregulated in microvascular ECs isolated from rat skeletal muscle exposed to mechanical stretch [282,283]. Also, mechanical stretch of the rat myocardium by expanding an intraventricular balloon or in response to hemodynamic overload via an aorto-caval shunt upregulates HIF-1α [284]. HIF-1α expression was also upregulated in response to cyclic stretch for 4 h in rat VSMCs [285] and for 24 h in rat Achilles tendon fibroblasts [286]. Mechanical stretch-induced HIF partly involves activation of PI_3_K and MAPK [284,285]. Also, in malignant glioma cell lines, HIF-1α could promote MMP-2 and MMP-9 expression and decrease TIMP-2 expression [287]. Interestingly, prolonged mechanical stretch of isolated rat inferior vena cava increases the expression of HIF-1α, and downstream MMP-2 and MMP-9 [288]. Plasma volume expansion and increased vascular stretch may upregulate vascular HIF and MMPs during Norm-Preg, and these mechanisms may be altered with reduced plasma volume in PE.

### 6.4. Reactive Oxygen Species (ROS)

ROS are metabolic byproducts containing highly reactive O_2_ and include superoxide anion (O_2_^•−^), hydrogen peroxide (H_2_O_2_) and hydroxyl ion (OH^−^). Norm-Preg is a state of mild oxidative stress due to increased maternal metabolism, placental metabolic activity and ROS production [289], which are normally counterbalanced by adequate levels of antioxidants [11]. In PE, extended periods of ischemia/hypoxia promote inflammation, vascular dysfunction, and oxidative stress [260], and the levels of antioxidants such as hemeoxygenase-1, hemeoxygenase-2, copper/zinc superoxide dismutase, glutathione peroxidase and catalase in blood cells and plasma may be too low to counterbalance the increased placental ROS generation [290]. ROS/antioxidants imbalance increases the formation of lipid peroxides and thromboxane A_2_ (TXA_2_) and further inhibits glutathione peroxidase activity in the placenta [29]. The total antioxidant capacity is also lower in PE versus Norm-Preg serum [291], with the reduced levels of most of the studied antioxidants more strongly associated with EO-PE than LO-PE [292]. PE women also showed decreased plasma levels of the antioxidant ascorbate and reduced brachial artery flow-mediated dilation that were improved upon administration of ascorbic acid, supporting a role of oxidative stress in EC dysfunction associated with PE [293]. Hemeoxygenase-1 levels are also reduced in the placenta of RUPP versus Norm-Preg rats [32].

Neutrophils and monocytes are major producers of ROS in PE. Compared to monocytes from Norm-Preg women, monocytes from PE women generate greater quantities of O_2_^•−^ and H_2_O_2_ and inflict more EC damage [294,295]. Importantly, during Norm-Preg neutrophils also produce NO, which can counteract the damaging effects of O_2_^•−^ on ECs. However, in PE, the generation of excess O_2_^•−^ scavenges most of the NO produced by neutrophils and forms peroxynitrite (ONOO^−^), thus decreasing NO bioavailability and exacerbating EC damage [295]. NADPH oxidase isoform NOX1, which catalyzes one-electron reduction of O_2_ to O_2_^•−^, is upregulated in PE placenta [296]. Also, treatment of HUVECs with PE serum not only increases the expression of NADPH oxidase subunit gp91^phox^ and O_2_^•−^ production, but also increases the expression of iNOS [297], generating excess NO that could increase ROS formation and EC injury. Interestingly, treatment of RUPP rats with iNOS inhibitors decreases BP, aortic tissue levels of ROS and NADPH-dependent generation of ROS [298]. Also, in DOCA-salt HTN-Preg rats increased O_2_^•−^ production by NADPH oxidase, formation of ONOO^−^, and degradation of biopterin (BH_4_), which catalyzes eNOS dimerization and activation, cause eNOS uncoupling, decreased NO bioavailability and EC dysfunction [299]. In support, treatment of DOCA-salt HTN-Preg rats with a BH_4_ such as sepiapterin reduced O_2_^•−^ and ONOO^−^ and improved aortic NO production and endothelium-dependent relaxation of mesenteric arteries [299].

Other oxidative stress biomarkers such as malondialdehyde and prostaglandin F_2α_ are elevated in PE serum at gestational weeks 10–14, causing oxidative damage to the placenta even before the onset of PE [300]. Higher plasma 8-isoprostane and aortic and placental ROS levels were also detected in RUPP versus Norm-Preg rats [257,298]. In first-trimester villous trophoblasts, excessive oxidative stress alters the expression of multiple miRs that could alter angiogenesis, apoptosis, immune response and inflammation, and lead to PE [301]. In trophoblast cells and HUVECs treated with cobalt chloride to simulate hypoxic conditions, the free radical scavenger edaravone inhibited sFlt-1 expression in trophoblast cells and protected against the decrease in vascular development and tube formation in HUVECs [302]. Elevated ROS could also alter uteroplacental and vascular MMP expression/activity and contribute to PE pathogenesis [30]. In a simulated placental ischemia/reperfusion model of first-trimester trophoblast HTR-8/SVneo cells, exposure to 1 h ischemia buffer and 24 h reperfusion increased ROS production and O_2_ consumption rate and decreased cell migration, proliferation, and invasion, and MMP-9 expression. Antioxidants reversed the deficits in migration and proliferation, while MMP-9 inhibition resulted in deficient invasion. These observations suggested that ischemia/reperfusion impairs trophoblast migration and proliferation via a ROS-dependent mechanism, and invasion via an ROS-independent loss of MMP-9 [303]. More research into how ROS may interact with MMPs in PE should be conducted.

### 6.5. Ang II and AT_1_R Agonistic Autoantibodies (AT_1_-AA)

Ang II is a major regulator of water and electrolyte homeostasis and BP. Ang II activates AT_1_R in VSMCs causing increases in [Ca^2+^]_c_, ROCK activity, vasoconstriction, vascular growth, and inflammation. Ang II also activates AT_2_R in ECs, stimulating eNOS, NO release, PGI_2_ production, and vasodilation. Although the plasma renin and Ang II levels are increased during Norm-Preg, the pressor response to Ang II is decreased likely due to downregulation of AT_1_R or upregulation of AT_2_R. However, at gestational weeks 23–26, the Ang II dose needed to induce a 20 mmHg increase in diastolic BP was lower in women who later developed PE compared with Norm-Preg women who maintained normal BP [304], suggesting increases in the sensitivity to Ang II even before overt manifestations of PE.

Measurements of Ang II levels and AT_1_R mRNA expression showed increases in chorionic villi and placenta of PE compared with Norm-Preg women [305,306]. Importantly, plasma hemopexin activity is elevated in Norm-Preg women from gestational week 10 onward, and hemopexin downregulates AT_1_R in human monocytes and reduces functional AT_1_R and Ang II-induced contraction of rat aorta, suggesting that decreased hemopexin activity may contribute to upregulation of AT_1_R and enhanced vasoconstriction in PE [307].

AT_1_-AA is a bioactive factor that activates AT_1_R and promotes vasoconstriction and VSMC growth. Serum AT_1_-AA levels are greater in PE than Norm-Preg women [226,308], and are even higher in severe PE and LO-PE [309,310]. Circulating AT_1_-AA levels are also increased in RUPP versus Norm-Preg rats [257,311,312,313]. Preg mice infused with AT_1_-AA show PE features including increased BP, proteinuria and plasma sFlt-1 levels [226]. Also, Preg rats infused with AT_1_-AA showed increased ET-1 levels in the placenta and renal cortex [314], and reduced ACh-induced dilation of renal interlobar arteries, which was prevented by endothelin type A receptor (ET_A_R) antagonist, suggesting an interplay between Ang II and ET-1 in the setting of EC dysfunction and HTN-Preg [315]. AT_1_-AA may contribute to reduced trophoblast invasion, increased BP, sFlt-1, sEng, ROS and cellular Ca^2+^, activation of coagulation tissue factor, collagen-induced platelet aggregation, blood hypercoagulability, thrombosis, adrenal gland damage, and decreased aldosterone release in PE [33,226,308,316]. Circulating AT_1_-AA may also cross the placenta into the fetal circulation, affecting placental development and contributing to FGR [104]. Treatment of cultured trophoblasts with IgG from PE women increases sFlt-1 [317]. Also, HUVECs treated with AT_1_-AA from PE women show increases in the activity of caspase-3 and caspase-8 and the release of lactate dehydrogenase, leading to EC apoptosis and necrosis [318]. Infusion of Preg rats with TNF-α also increases plasma AT_1_-AA levels, suggesting a link between inflammatory cytokines and AT_1_-AA release in PE [313].

## 7. Aberrant Vascular Signaling and Remodeling in HTN-Preg and PE

**Figure 4:**
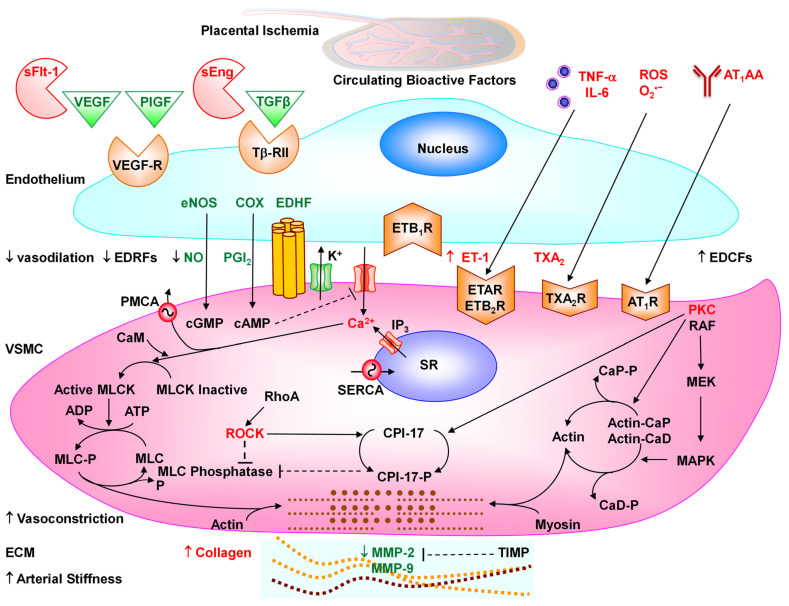
EC and vascular dysfunction in HTN-Preg and PE. Placental ischemia causes systemic release of sFlt-1 which antagonizes VEGF and PlGF, and sEng which antagonizes TGFβ, as well as TNF-α, IL-6, ROS, and AT_1_-AA. The bioactive factors target ECs, causing decreases in endothelium-derived relaxing factors (EDRFs) NO, PGI_2_ and EDHF and leading to decreased vasodilation. The circulating factors also increase the release of endothelium-derived contracting factors (EDCFs), ET-1 and TXA_2_, and activate ET_A_R, TXA_2_R and AT_1_R in VSMCs causing increases in cytosolic Ca^2+^, MLC phosphorylation, activation of PKC, MEK, MAPK and ROCK, and increases in Ca^2+^ sensitivity of the contractile proteins, leading to increased vasoconstriction. The bioactive factors also target ECM causing MMPs/TIMPs imbalance, and collagen accumulation, leading to increased arterial stiffness. CaD, caldesmon; cAMP, cyclic adenosine monophosphate; CaP, calponin; CPI-17, PKC-potentiated inhibitor protein of 17kDa; MEK, MAPK; MLCK, myosin light chain kinase; PMCA, plasmalemmal Ca^2+^-ATPase; SR, sarcoplasmic reticulum; SERCA, SR Ca^2+^-ATPase.

Circulating bioactive factors target different components of the blood vessels including ECs, VSMCs and ECM causing changes in various vascular signaling pathways and vascular remodeling by MMPs and leading to HTN-Preg and PE (Figure 4).

### 7.1. EC Dysfunction in PE

Healthy and functional endothelium is essential to maintain Norm-Preg and favorable outcomes for the Preg woman and the fetus [319]. Norm-Preg is associated with increases in brachial artery flow-mediated dilation [320]. Bradykinin induces a greater endothelium-dependent relaxation in small subcutaneous arteries from Preg versus non-Preg women [321]. Also, studies showed more frequent periodic [Ca^2+^]_c_ bursts in uterine artery ECs from Preg compared with non-Preg ewes [322], which would stimulate the release of Ca^2+^-dependent vasodilator factors, and enhance uteroplacental blood flow during Norm-Preg [323].

Gonadal and placental hormones contribute to the pregnancy-related changes in the hemodynamics and vascular function. E2 stimulates the release of NO, PGI_2_ and EDHF and promotes endothelium-dependent vasodilation [324]. E2 also induces endothelium-independent relaxation in endothelium-denuded vessels by decreasing [Ca^2+^]_c_ and inhibiting PKC-mediated Ca^2+^ sensitization mechanisms of VSMC contraction [38,325,326,327]. Additionally, E2 could modulate the vascular cytoskeleton and ECM [324]. The vasodilator effects of E2 partly involve increases in MMP expression/activity. In human coronary artery and umbilical artery VSMCs, E2 increases MMP-2 release in a concentration-dependent manner [47]. P4 promotes the vasodilator effects of E2 by activating similar mechanisms [35,324,327].

PE is associated with systemic EC dysfunction and HTN, glomerular endotheliosis leading to renal injury and proteinuria, and cerebral endotheliosis causing cerebral edema and seizures [11]. Brachial artery flow-mediated dilation is reduced in PE versus Norm-Preg women [319,328]. Also, PE women show decreased flow-mediated radial artery dilation (~7.9%) compared with Norm-Preg women (~17.4%) [329]. Bradykinin causes less relaxation in small subcutaneous arteries of PE versus Norm-Preg women [321]. Also, markers of endothelial injury/damage such as circulating ECs, soluble VCAM-1, endocan and E-selectin are elevated [216,250,330,331], while progenitor ECs are decreased in PE versus Norm-Preg women [332].

Copeptin, a marker of arginine vasopressin secretion, is elevated in PE. Also, AVP infusion in Preg mice induces HTN-Preg, glomerular endotheliosis, placental oxidative stress and FGR, and decreases PlGF [333]. Importantly, RUPP rats, which show many features of PE including elevated BP, proteinuria, decreased glomerular filtration rate and renal plasma flow, and FGR, have been useful in characterizing the mechanisms of vascular dysfunction in HTN-Preg and PE [24,334,335]. ACh is less potent in producing relaxation in the aorta and mesenteric microvessels of RUPP versus Norm-Preg rats, suggesting EC damage/dysfunction [23,335]. ECs also release contracting factors such as ET-1 and TXA_2_, and EC dysfunction causes imbalance in vasodilator versus vasoconstrictor substances.

#### 7.1.1. Changes in Nitric Oxide (NO) in PE

NO is a major vascular relaxing factor and vasodilator. NO diffuses into VSMCs, activates guanylate cyclase and increases cGMP, which stimulates Ca^2+^ efflux through plasmalemmal Ca^2+^-ATPase, decreasing [Ca^2+^]_c_ and causing vascular relaxation. Nitrites are NO metabolites that show higher plasma levels in Preg versus non-Preg women [336]. Also, the plasma and urinary levels of cGMP are elevated in Norm-Preg. Additionally, NOS expression and activity in human uterine artery and placenta are augmented with gestational age [337,338].

Polymorphism in the *eNOS* gene affects NO production and contributes to the risk of PE. In a study of Preg Brazilian women, *eNOS* gene polymorphisms VNTRa and 894T were associated with severe EO-PE and LO-PE, respectively. Women with *eNOS* VNTRb/a polymorphism and the aa allele showed lower levels of plasma NO metabolites. Also, women homozygous for the eNOS 894T allele were more prone to selective proteolytic degradations in vascular ECs and tissues, leading to decreased NO production [339]. The eNOS polymorphism T786C was also observed more frequently in PE than Norm-Preg women [340,341]. Interestingly, for the T786C allele, plasma nitrite levels were lower in Preg women with the TT phenotype than the CC phenotype [342], and the TT phenotype has been associated with increased risk of PE among Tunisian women [340].

EC dysfunction causes decreases in NO synthesis/release [343]. However, measurements of plasma nitrite levels varied from increases [344] to decreases [345,346,347,348] in PE versus Norm-Preg women. Measurements of urinary nitrites also showed no difference in PE compared with Norm-Preg women [345]. The discrepancies in the measurements of nitrite levels may stem from the difficulties in controlling dietary nitrate. However, a careful study that controlled nitrate/nitrite dietary intake did not find a decrease in whole-body NO production among PE women [349], suggesting tissue-specific alterations in NOS expression/activity and NO release [11]. For instance, nitrite levels were decreased in the placenta of PE versus Norm-Preg women [348]. Also, compared with Norm-Preg women, eNOS mRNA expression was lower in the umbilical cord of PE women [350] and even lower in severe PE [351,352]. Conversely, one study found an increase in eNOS mRNA in the placenta of PE women [353]. Also, although cGMP plasma and urinary levels are elevated in Norm-Preg, they do not show marked differences in PE [345].

Experimental studies support pregnancy-related increases in NO production. Urinary nitrate/nitrite and renal iNOS and nNOS protein levels were elevated, although renal eNOS decreased, in Preg versus virgin rats [354]. Preg rats supplemented with the NOS blocker L-NAME in the drinking water show PE-like manifestations including elevated BP, renal vasoconstriction, proteinuria, thrombocytopenia and FGR [36,224,355]. However, measurements of plasma nitrites were not different in L-NAME-treated versus nontreated Preg rats [224]. Compared with Norm-Preg rats, RUPP rats showed reduced plasma nitrite levels [334], but no differences in urinary nitrite levels [24,356]. Also, as in humans, no differences in circulating levels of the NOS substrate L-arginine were found in RUPP versus Norm-Preg rats [357]. On the other hand, vascular function studies showed increased aortic contraction to the α-adrenergic agonist phenylephrine in L-NAME-treated Preg rats [36]. Also, eNOS expression and ACh-induced nitrates/nitrites production were reduced in the mesenteric artery and aorta of RUPP compared with Norm-Preg rats, supporting tissue-specific decreases in NO production [23,334,335]. DOCA-salt HTN-Preg rats also show decreases in ACh-induced NO-dependent relaxation of mesenteric vessels, although mRNA expression of eNOS is increased [299]. One study showed increased MMP-2 and MMP-9 activity in the uterus, placenta, and aorta in association with reduced NO levels and increased oxidative stress in L-NAME-treated versus non-treated Preg-rats, suggesting that NO bioavailability could differentially regulate MMP activity in Norm-Preg and HTN-Preg [358]. On the other hand, MMP-induced proteolytic degradation may affect eNOS enzyme activity and NO production by ECs, making it important to further examine the relation between MMPs and NO synthesis in PE.

#### 7.1.2. MMPs and EDHF

EDHF is an important relaxing factor that controls the diameter of small microvessels, regional blood flow, vascular resistance and BP. In ECs, activation of EDHF promotes K^+^ efflux through intermediate (IK_Ca_) and small conductance Ca^2+^-activated K^+^ channels (SK_Ca_) causing membrane hyperpolarization, which propagates to neighboring VSMCs via myoendothelial gap junctions (MEGJs) and connexins causing VSMC hyperpolarization, inhibition of Ca^2+^ influx through Ca^2+^ channels and VSMC relaxation. The opening of EC IK_Ca_ and SK_Ca_ and accumulation of K^+^ between ECs and VSMCs could also activate inwardly rectifying K^+^ (K_IR_) channels and Na^+^/K^+^-ATPase, causing further VSMC hyperpolarization [359]. Other diffusible EDHFs include the cytochrome P450 (CYP450) product epoxyeicosatrienoic acid (EET), and H_2_O_2_ which activate large conductance K_Ca_ (BK_Ca_), and promote relaxation of VSMCs [360].

In small subcutaneous and myometrial arteries from Norm-Preg women, bradykinin-induced relaxation is mediated by both EDHF (50%) and NO to maintain appropriate vascular tone [361,362]. Also, the gap junction proteins connexins 37, 40 and 43 facilitate EDHF-mediated relaxation of omental arteries and veins from Norm-Preg women [363]. In uterine radial arteries of Preg rats, increases in EC [Ca^2+^]_c_ could activate IK_Ca_ and SK_Ca_, promoting K^+^ efflux and vascular relaxation [364]. EDHF-mediated relaxation of Preg rat uterine vessels may also involve activation of a delayed rectifier type of voltage-sensitive K^+^ channels (K_v_) [365].

While MEGJs alone could be the main EDHF-mediated relaxation pathway in subcutaneous arteries from Norm-Preg women, they are assisted with H_2_O_2_ or CYP450 epoxygenase metabolites of arachidonic acid to mediate EDHF-induced dilation in blood vessels of PE women. This is likely due to morphological changes in the blood vessel wall and consequent diminishing of MEGJs’ role in PE [366]. Experimental studies also show decreased reactivity to phenylephrine and augmented bradykinin-induced vasodilation in blood vessels of Preg WT mice, but not in KO mice deficient in nuclear pregnane X receptor and CYP450 expression. Also, bradykinin-induced vascular relaxation is markedly blocked by CYP450 inhibitor in WT but not pregnane X receptor KO mice, indicating a role of EET and other CYP450 metabolites in pregnancy-related vascular adaptation [367]. These observations suggest that impaired vascular relaxation in HTN-Preg partly involves changes in EDHF. However, EDHF-mediated relaxation was not different in mesenteric microvessels of RUPP compared with Norm-Preg rats [335]. Given that impaired EDHF-mediated vasodilation is a major factor in vascular resistance and systemic HTN, further studies should examine its role in PE.

Our studies on rat veins have shown that prolonged increases in venous pressure/wall tension are associated with increases in the expression of MMP-2 and MMP-9 [52,53,54]. Also, MMP-2 and MMP-9 inhibit phenylephrine-induced contraction in rat inferior vena cava partly through membrane hyperpolarization and K^+^ channel activation [53,54]. It is plausible that pregnancy-related expansion of plasma volume and intravascular stretch could increase MMP-2 and MMP-9 expression, causing vasodilation and decreased BP. On the other hand, decreases in MMP-2 and MMP-9 expression and vascular relaxation pathways may participate in the enhanced vasoconstriction and elevated BP observed in HTN-Preg and PE [23,335,368].

#### 7.1.3. MMPs and Endothelin-1 (ET-1) in PE

ET-1 is an EDCF with a major role in some forms of HTN including HTN-Preg [369,370,371,372,373,374,375,376,377]. PreproET is cleaved by furin-like protease to biologically inactive big-ET, which is further cleaved by endothelin converting enzymes into active ET-1. PE-related bioactive circulating factors could induce ET-1 release from ECs [376]. In support, HUVECs produce larger amounts of ET-1 when treated with serum from PE than Norm-Preg women [378]. Studies also showed marked elevation of plasma ET-1 levels at the later stages of PE and their reversal to normal levels within 48 h postpartum [379,380], indicating a role for ET-1 in the progression rather than the initiation of PE. Other studies showed no differences in serum ET-1 levels in PE compared with Norm-Preg women, and found elevated ET-1 levels mainly in women with HELLP syndrome [182,381,382]. Given that ECs release ET-1 in a directional paracrine fashion toward VSMCs, PE-related elevation of ET-1 levels could be localized in blood vessels and other tissues. In support, a 4–8-fold increase in ET-1 levels was detected in HUVECs exposed to low compared to normal O_2_ tension [383]. Also, placentae exposed to hypoxia release large amounts of ET-1 from both the maternal and fetal side [384]. Angiogenic imbalance could contribute to the changes in ET-1 levels. PE women with sFlt-1/PlGF ratio > 85 have higher plasma levels of ET-1 than women with sFlt-1/PlGF ratio < 85 [385]. Also, mice treated with sFlt-1 show increased vascular response to ET-1 [386]. Additionally, RUPP rats show increases in preproET levels by 45% in renal cortex and 22% in renal medulla [377]. While circulating ET-1 levels may not reflect the local tissue levels in mild PE, during severe PE and HELLP syndrome EC ET-1 release could be so high such that it loses its paracrine directionality and spills over to the entire circulation. In support, rat models of severe PE and HELLP syndrome show increases in plasma ET-1 levels [244,387].

ET-1 contributes to PE pathogenesis by promoting trophoblast apoptosis and the release of anti-angiogenic and oxidant substances [376,388]. In VSMCs, ET-1 activation of ET_A_R stimulates Ca^2+^ release from the sarcoplasmic reticulum, Ca^2+^ influx through Ca^2+^ channels, and PKC-mediated inhibition of K^+^ channels, causing increases in [Ca^2+^]_c_ and VSMC contraction [371,389,390,391,392,393,394]. Preg rats show decreases in ET-1-induced constriction in mesenteric vessels and ET_A_R levels in the aortic media and VSMCs [395,396]. Also, BP is reduced in RUPP and other HTN-Preg rats treated with ET_A_R antagonists [369,377,397]. Interestingly, 52% of Brazilian women with PE have severe PE and high levels of ET_A_R agonistic autoantibodies (ET_A_-AA), causing ET_A_R activation and severe vasoconstriction [398].

ET-1 also activates ET_B_R in ECs, inducing the release of NO, PGI_2_ and EDHF, and promoting renal artery dilation and hyperfiltration in Preg rats [389,399]. Decreased ET_B_R expression may impair trophoblast invasiveness in PE. ET_B_R antagonists also decrease vasodilation in microvessels of Preg rats, and ET_B_R levels are reduced in ECs and renal cells of RUPP rats. Also, ET_B_R-mediated NO release is reduced in the aorta and mesenteric artery of RUPP versus Norm-Preg rats, suggesting downregulation of EC ET_B_R in HTN-Preg [335].

Because of their proteolytic activity, MMPs can break big-ET-1 into smaller endothelin peptides with different affinities to ET_A_R and ET_B_R and varying effects on vascular reactivity in Norm-Preg and PE. MMP-induced degradation of big-ET into ET-1 could activate ET_A_R and enhance vasoconstriction [400]. In omental vessels from Norm-Preg women, MMP-1 induces vasoconstriction and enhances Ang II-induced constriction likely through activation of endothelial protease-activated receptor-1 (PAR-1) and ET-1 release [144]. ET-1 could then activate ET_A_R-mediated mechanisms of VSMC contraction [371,394,401] including [Ca^2+^]_c_ [402,403,404], PKC [405,406,407,408,409,410,411], and ROCK [412,413,414]. However, MMP-2 and MMP-9 could degrade big-ET into ET_1–32_ which preferentially activates EC ET_B1_R and promotes vascular relaxation. Preg rats show increases in vascular MMP-2, MMP-9, and ET_B_R [48,395,415]. Also, consistent with the observed reduction in vascular MMP-2 and MMP-9 levels, RUPP rats show decreases in ET_B_R levels, and infusing Preg rats with the ET_B_R antagonist BQ788 causes increases in BP [335].

### 7.2. VSMC Dysfunction in PE

#### 7.2.1. VSMC Ca^2+^ in PE

Ca^2+^ promotes VSMC contraction and growth. Initial Ca^2+^ release from the sarcoplasmic reticulum and maintained Ca^2+^ influx through Ca^2+^ channels increase VSMC [Ca^2+^]_c_. Ca^2+^ binding and formation of Ca^2+^-calmodulin complex activates myosin light chain kinase, and increases myosin phosphorylation, actin–myosin interaction and VSMC contraction. Decreased [Ca^2+^]_c_ dissociates the Ca^2+^-calmodulin complex, and activates myosin phosphatase, leading to myosin light chain dephosphorylation and VSMC relaxation. EDRFs activate different Ca^2+^ removal pathways and decrease VSMC [Ca^2+^]_c_. Norm-Preg is associated with increases in K_Ca_ channel activity leading to VSMC hyperpolarization, decreased uterine artery tone and increased uteroplacental blood flow. Hypoxia decreases EDRFs and VSMC Ca^2+^ extrusion mechanisms, but increases EDCFs, thus augmenting [Ca^2+^]_c_ and VSMC contraction. In a sheep model of gestational hypoxia, decreased uterine artery K_Ca_ channel activity augments VSMC [Ca^2+^]_c_ and vasoconstriction leading to insufficient uteroplacental blood flow [416]. Notably, myometrial vessels from Norm-Preg and PE women showed no difference in their vasoconstriction to high KCI, phenylephrine or Ang II [417]. However, basal and agonist-induced [Ca^2+^]_c_ were greater in renal arterial VSMCs of L-NAME-treated versus non-treated Preg rats [418]. Also, Ang II via AT_1_R causes marked increases [Ca^2+^]_c_ in the platelets, erythrocytes, and lymphocytes of PE women, but little increases 6 weeks postpartum [316]. Ang II- and caffeine-induced contraction and transient [Ca^2+^]_c_ in Ca^2+^-free medium are similar in VSMCs, while KCl-induced maintained [Ca^2+^]_c_ in a Ca^2+^-containing solution is greater in renal arterial VSMCs of RUPP versus Norm-Preg rats, indicating increases in Ca^2+^ channel activity rather than Ca^2+^ release from the intracellular stores [419].

Interestingly, plasma of non-Preg and PE women, but not Norm-Preg women, enhanced myogenic tone and blunted methacholine-induced relaxation in mesenteric arteries of non-Preg female mice, and the MMP inhibitor GM6001 surprisingly further enhanced myogenic tone and abrogated relaxation particularly in vessels treated with PE plasma. These observations suggest altered adaptation to pregnancy by MMPs in the vasculature of PE compared to Norm-Preg women [420]. Studies on rat veins have suggested that MMP-2 and MMP-9 may induce VSMC hyperpolarization, leading to decreased Ca^2+^ entry and vascular relaxation [52,53,421], and a decrease in these MMPs could promote Ca^2+^ influx, vasoconstriction and HTN-Preg.

#### 7.2.2. PKC, MAPK, and ROCK in PE

PKC contributes to maintaining VSMC contraction by phosphorylating PKC-potentiated inhibitor protein of 17 kDa (CPI-17) which inhibits myosin phosphatase and leads to increased myosin light chain phosphorylation. PKC can also phosphorylate the actin-binding protein calponin, thus facilitating actin–myosin interaction and augmenting VSMC contraction. Phorbol esters activate PKC and cause VSMC contraction without changing [Ca^2+^]_c_, mainly through increasing the myofilaments’ force sensitivity to Ca^2+^. Uterine artery of Preg ewes and gilts and the aorta of Preg rats show decreased PKC activity and vascular contraction [422,423,424]. Also, the protein levels, membrane translocation and activity of Ca^2+^-dependent α-PKC and Ca^2+^-independent δ- and ζ-PKC are lower in aortic VSMCs of Preg versus non-Preg rats, but greater in L-NAME-treated versus non-treated Preg rats [423,425]. Studies also implicate PKC in the increased AT_1_-AA levels and vascular AT_1_R activity in PE. In support, in cultured rat cardiomyocytes, IgG isolated from PE women enhances AT_1_R-mediated contraction that is reversed by the PKC inhibitor calphostin C [426]. In ovine uterine arteries, PKC-mediated contraction is reduced due to upregulated BK_Ca_ channel during Norm-Preg, while PKC is upregulated and inhibits BK_Ca_ during gestational hypoxia [416]. PKC inhibitor bisindolylmaleimide-I reduced TXA_2_ analog U46619-induced contraction in uterine and mesenteric arteries of non-Preg and Preg rats [427]. Also, cicletanine inhibits PKC and lowers BP in HTN-Preg rats. In the aorta of endotoxemic rats, MMP-2 may reduce contraction partly through degrading the PKC target and actin-binding protein calponin [428], and a decrease in MMP-2 would prevent calponin proteolysis and allow it to promote VSMC contraction in HTN-Preg.

MAPK regulates cellular gene expression, mitosis, and differentiation. Also, during maintained VSMC activation, PKC causes phosphorylation of MAPK and subsequent phosphorylation/activation of MAPK, leading to phosphorylation of the actin-binding protein caldesmon and enhancement of VSMC contraction. Changes in MAPK have been linked to vascular remodeling in pathological conditions such as atherosclerosis [429], and PKC-mediated MAPK/caldesmon phosphorylation could alter VSMC contraction and modulate vascular function during Norm-Preg and HTN-Preg. In support, inhibition of ERK_1/2_ or p38 MAPK decreased TXA_2_ analog U46619-induced contraction in uterine and mesenteric arteries of Preg and non-Preg rats [427].

ROCK regulates cell migration, cytoskeletal reorganization and VSMC contraction. ROCK-I (ROKβ) and ROCK-II (ROKα) facilitate the formation of placental microvilli during Norm-Preg, while placental villi from PE women show abnormal ROCK-II expression and apoptosis of the syncytium [430]. Also, subcutaneous microvessels from PE women show increases in ROCK activity and Ca^2+^ sensitivity of the contractile myofilaments [431]. In L-NAME-treated HTN-Preg rats, Ang II may activate AT_1_R and the cardiovascular RhoA/ROCK pathway [432]. Also, IL-17 through activation of ROCK promotes phosphorylation of Thr495 and inhibition of eNOS, leading to decreased NO production and increased BP in mice [433]. On the other hand, ROCK inhibitor Y-27632 decreases TXA_2_ analog U46619-induced contraction in the uterine artery of non-Preg rats [427]. Also, the ROCK inhibitor fasudil attenuated sFlt-1 induced HTN in Preg mice [434]. Other studies found a decrease in ROCK expression in the umbilical artery of PE women [435], prompting further investigation into the role of ROCK signaling in various vascular beds.

MMP-induced proteolysis of big-ET and subsequent ET-1 release and activation of VSMC ET_A_R could activate PKC, MAPK and ROCK-mediated pathways, leading to increases in the Ca^2+^ sensitivity of contractile proteins, enhanced vasoconstriction, and HTN-Preg.

### 7.3. MMPs and ECM Protein Degradation in PE

MMPs proteolyze multiple ECM protein substrates such as collagen [9,10,436], thus promoting ECM degradation and facilitating cell growth and migration. Our research showed enhanced Picro-Sirius Red staining and a greater collagen content in uterus, placenta and aorta of RUPP versus Norm-Preg rats [8]. The decreased MMPs and increased collagen in RUPP rat tissues could impede cell growth, proliferation and migration, and interfere with uteroplacental remodeling. Despite the increased collagen content of the RUPP rat aorta, there was a decrease in aortic tissue weight and thickness, likely because of impeded VSMC growth. In support, MMP-2 or MMP-9 KO in a mouse model of neointimal hyperplasia produced by carotid artery ligation/occlusion/injury was associated with decreased VSMC migration/proliferation, and neointima formation [437,438,439,440]. Decreased vascular MMP activity and increased collagen content could also decrease blood vessel plasticity and increase vascular rigidity, thus augmenting vascular resistance and HTN. In support, decreases in MMP-1, MMP-2, and MMP-9 activity and increases in collagen content were observed in the internal mammary artery from hypertensive compared with normotensive patients undergoing coronary artery bypass surgery [441]. Also, in mice receiving high salt diet and Ang II infusion to elevate systolic BP, MMP-9 KO further promotes vascular stiffness and increases pulse pressure, suggesting a role of MMP-9 in preserving vessel compliance and reducing BP in early HTN [442]. Decreases in MMP-1, MMP-2, and MMP-3 may also contribute to remodeling of resistance vessels and the setting of HTN in spontaneously hypertensive rats [443]. We have also reported increases in Ca^2+^-dependent intrinsic tone, remodeling, and arterial stiffness with augmented MMP-1 and MMP-7 levels in mesenteric microvessels and uterine arteries of RUPP rats [444,445]. Collagen has 18 types and different subtypes [446]. MMP-2 degrades collagen I, II, III, IV, V, VII, X, and XI while MMP-9 degrades collagen IV, V, VII, X and XIV [9,10,436]. Importantly, collagen-I and IV were abundant in the aorta, uterus, and placenta of Preg + sFlt-1, Preg + TNF-α, and RUPP rats, and were decreased in RUPP + PlGF and RUPP + etanercept rats [237,262]. Studies should examine various collagen subtypes in HTN-Preg and PE and use RT-PCR analysis to test if increased collagen levels are due to reduced proteolytic degradation or diminished de novo mRNA expression and protein biosynthesis.

In contrast with collagen, elastin staining in tissue sections of the uterus and placenta was sparse, less defined and not different in RUPP versus Norm-Preg rats, suggesting a little role in uteroplacental growth and remodeling. Also, while the aorta showed prominent and clearly defined elastin bands, no marked changes could be detected between RUPP and Norm-Preg rats, indicating a minimal role of elastin in the observed aortic and vascular remodeling [8].

Although MMPs are well-recognized for their proteolytic effects on ECM proteins, they could activate other cellular and molecular pathways that ultimately affect uteroplacental and vascular function [52,53,144,415]. MMP-2 and MMP-9 induce relaxation of rat aorta and inferior vena cava precontracted with phenylephrine [52,53,54], and altered vascular expression/activity of these MMPs may change EC and VSMC function, decrease vasodilation, and increase vasoconstriction in HTN-Preg and PE.

MMPs comprise a large family of at least 28 enzymes with different proteolytic properties [9,10,436]. In addition to MMP-2 and MMP-9, other MMPs are expressed in the uterus, placenta and blood vessels. In cultured rat aortic VSMCs, interstitial flow induced MMP-1 expression and cell migration via an ERK1/2-dependent and c-Jun-mediated mechanism, suggesting a role of MMP-1 in VSMC migration and neointima formation following vascular injury [447]. Also, endogenous MMP modulators influence MMP activity. For instance, MT1-MMP can cleave and transform inactive proMMP-2 to active MMP-2 [9,10,436,448]. Also, the macrophage metalloelastase MMP12 is secreted by trophoblasts and may play a role in elastin degradation in the spiral arteries during the initial placentation stages [147,148]. Notably, MMP-12 activates proMMP-2 and proMMP-3, which can in turn activate proMMP-1 and proMMP-9, thereby triggering a proteolysis cascade that degrades various ECM proteins [148]. NF-κB is also an important MMP modulator positively correlated with secretion of MMP-2 and MMP-9 during ECM disorganization, trophoblast invasion and spiral arteries remodeling [449,450]. MMP activity is also influenced by endogenous inhibitors such as TIMPs [9,10,436]. For example, TIMP-2 is as effective as specific MMP-2 blocking antibody in inhibiting MMP-2 and reducing the invasive capacity of cultured trophoblasts [46]. We have also found a decrease in MMP-9 homodimerization and an increase in its complexation with TIMPs in the uterus of RUPP versus Norm-Preg rats [451], making it important to further examine the role of MMPs in modulating the intrauterine environment in HTN-Preg and PE. Also, the time course and the progressive changes in MMPs with gestational age, EO-PE and LO-PE, mild and severe PE, and during the postpartum period warrant further discussion.

## 8. Localized Effects of Uteroplacental MMPs and FGR

**Figure 5:**
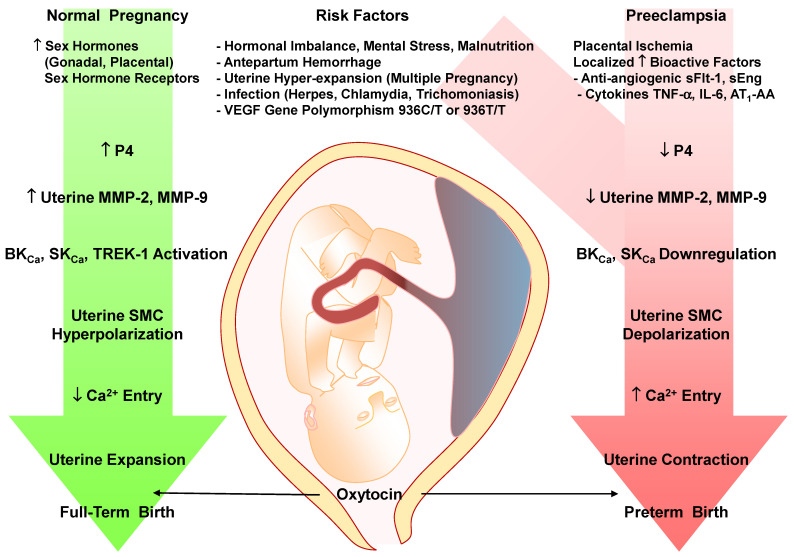
Factors influencing uterine function in Norm-Preg and PE. During Norm-Preg, increases in P4 levels cause increases in uterine MMP-2 and MMP-9 expression/activity, leading to activation of K^+^ channels, uterine SMC hyperpolarization, and uterine expansion to accommodate the growing fetus until full-term birth. PE and other risk factors that complicate pregnancy could decrease P4, leading to decreased uterine MMP-2 and MMP-9 expression/activity, K^+^ channels downregulation, increased uterine SMC depolarization, uterine contraction, and preterm birth. Oxytocin facilitates cervical dilation during the initial stages, then induces uterine contraction in the second and third stages of labor.

During PE development, placental ischemia promotes the release of bioactive factors not only systemically into the maternal circulation, but also locally within the uteroplacental tissues, causing changes in the expression/activity of MMPs, further decreases in trophoblast invasion of spiral arteries, and inadequate uteroplacental remodeling, thus interfering with physiological uterine function and fetal growth, and leading to FGR and preterm birth (Figure 5).

### 8.1. MMPs and Uterine Function

The uterus responds differently to mechanical and chemical stimuli in the non-Preg, Norm-Preg, and parturition states. In the non-Preg uterus, transient mechanical stretch causes myogenic contraction, while during Norm-Preg, a balance between uterine contraction and relaxation mechanisms reduces myometrium excitability and maintains a quiescent state until full-term [452]. Preg-related adaptations include uterine SMC hypertrophy/distension and extensive ECM remodeling, leading to progressive uterine expansion that allows sufficient space for the fetus to grow [453,454,455]. At full-term, activation mechanisms increase myometrium excitability, stimulate uterine SMC contraction, and initiate normal labor. Premature activation of the uterine contractile mechanisms disrupts uterine quiescence and causes preterm birth.

MMPs play an important role in regulating uterine function and endometrial tissue remodeling during the estrous and menstrual cycles and during pregnancy [40,41,42,456,457]. Changes in MMP-2, MMP-7 and MMP-9 expression also contribute to endometrial pathology in menstrual cycle disorders and endometriosis [458,459]. MMP-2 and MMP-9 are expressed in uterine NK cells during early pregnancy [460], and MMP-2 expression is increased during the final cervical ripening stages and collagen denaturation in late pregnancy [461]. MMP-2, MMP-7 and MMP-9 have also been detected in the rat and bovine uterus [48,415,462,463], and in the bovine endometrium and myometrium throughout gestation [40,457,463]. MMP-2 and MMP-9 are also upregulated in the myometrium of late-Preg rats [415], where they enhance ECM degradation and promote uterine and cervical remodeling [464].

Among several biophysical and biological factors, mechanical stretch, and neurohypophysial and sex hormones can regulate SMC function and MMP expression/activity. Skeletal muscle fibers exposed to mechanical stretch show increases in MMP-2 expression [283]. Also, protracted stretch of rat inferior vena cava increases MMP-2 and MMP-9 expression and decreases vein contraction [53,54,421]. Likewise, uterine distension/wall stretch induced MMP-2 and MMP-9 upregulation and led to uterine relaxation, which were reversed by MMP inhibitors [415]. Along with mechanical stretch and changes in uterine MMPs, oxytocin is a neurohypophysial hormone released by the hypothalamus-pituitary as well as the adrenal medulla [465], corpus luteum [466,467], and placenta [468]. Plasma oxytocin levels are very low in the non-Preg state and during the menstrual cycle, but increase in gestational week 12 and progressively with gestational age [469]. Oxytocin facilitates cervical dilation during the initial stages of labor, then induces uterine contraction in the second and third stages. We showed that oxytocin-induced contraction was reduced in myometrium strips of mid- and late-Preg versus non-Preg rats [415]. Other studies showed greater oxytocin contraction in mid- and late-Preg than non-Preg rat uterus [470], as well as increases in mRNA expression of oxytocin, prostaglandin F2α, and ET-1 receptors and the density of α1-adrenergic receptors in correlation with E2 levels in late-Preg rats [470,471]. The discrepancy is likely caused by the Preg uterus being bigger and thicker such that measuring uterine contraction in absolute grams could show increases, while normalizing the contraction to uterine weight would show decreases, confirming pregnancy-related decrease in uterine SMC reactivity. Importantly, MMP-2 and MMP-9 expressions/levels were greater in Preg versus non-Preg rat uterus [48]. Also, the reduced uterine contraction in Preg rats was reversed by MMP inhibitors [415], supporting that prolonged uterine wall stretch during pregnancy causes upregulation of MMP-2 and MMP-9, which promote uterine expansion. MMP-7 (matrilysin-1) was also detected in the human uterus, amniotic fluid and fetal membranes, and its levels increased progressively during gestation [472]. Conversely, we found decreases in MMP-7 expression in Preg-rat uterus [415], suggesting different mechanisms regulating MMP-7 compared with MMP-2 and MMP-9 in different species and in the myometrium versus fetal membranes.

Steroid hormones also influence uterine structure and function during the menstrual cycle, pregnancy and parturition [473]. Plasma E2 levels increase during the proliferative phase of the menstrual cycle, then a decrease in E2 initiates endometrial shedding, and an increase in P4 promotes the luteal phase [474]. E2 and P4 regulate myometrial growth and contractility via both genomic and non-genomic pathways. Also, pregnancy is associated with marked increases in plasma E2 and P4 levels [37,471], further contributing to progressive uterine expansion [415,475,476,477]. While E2 may have stimulatory effects on uterine contraction at the time of parturition, these effects are neutralized during the course of pregnancy, likely due to decreased uterine expression of E2 receptors [478]. Also, E2 causes rapid non-genomic relaxation of spontaneous and depolarization-induced contraction of non-Preg rat uterus [475]. In comparison, P4 consistently causes uterine relaxation in the luteal phase and during pregnancy [478], which maximizes uterine receptivity to embryo implantation and IVF [479]. In support, endometrial P4 receptors are upregulated during early pregnancy [474]. Importantly, during IVF, application of vaginal P4 gel 2 days prior to embryo transfer promotes uterine relaxation and embryo implantation and permanence in the endometrium [476]. Also, in women aged 25 to 41, a 2-week transdermal E2 to simulate its levels in the late follicular phase, and vaginal application of P4 gel every two days from cycle day 15 decreased uterine contraction [480]. Similarly, P4 reduces contraction in non-Preg rat uterus [415].

E2 and P4 influence endometrium shedding in the menstrual cycle, the course of endometrial lesions, and uterine remodeling during pregnancy partly through changes in MMPs [456,481,482,483]. E2 promotes activator Protein-1 (AP-1) transcription, c-Jun and c-Fos, which in turn enhance MMP-2 and MMP-9 expression [484]. Also, in porcine endometrial explants, E2 induced MMP-8 and MMP-12 expression, while P4 decreased MMP-12 expression [267]. In mouse uterus, E2 or E2 + P4 increased MMP-9 activity, although E2 decreased MMP-9 mRNA expression, while MMP-2 expression/activity was not affected [456]. On the other hand, E2 and P4 increased MMP-2 and MMP-9 levels in the non-Preg rat uterus [48,415,481]. Also, serum MMP-2 and MMP-9 activity was higher in Preg than non-Preg bitches and correlated with serum E2 levels [63]. E2 could enhance uterine MMP levels through E2 receptor-mediated activation of MAPK [485]. E2 may also increase TNF-α and IL-6, which in turn affect MMP expression [456,485,486,487,488]. Other studies showed that P4 reduced BP, urine protein and plasma TNF-α and IL-1β levels, and increased IL-4, IL-10, IL-13, cyclin D1, and proliferating cell nuclear antigen (PCNA) in PE women. Also, in L-NAME-treated rat model of HTN-Preg, P4 improved PE-like features, reduced BP and urine protein, widened uterine spiral arteries, reduced inflammation, fibrinoid necrosis of the uterine wall and atherosclerotic lesions, and activated PI_3_K/AKT signaling pathway. Additionally, P4-enhanced proliferation and invasion, and increased the levels of MMP-2, MMP-9, p-AKT, and p-PI_3_K in HTR-8/Svneo cells, which were reversed by PI_3_K inhibitor LY294002, supporting P4-induced regulation of trophoblast cells through activation of MMPs and PI_3_K/AKT signaling [489]. The MMP inducer EMMPRIN could also affect uterine MMP expression during pregnancy [41,64,463,490]. EMMPRIN, MMP-2 and MMP-9 were upregulated in the uterus and aorta of mid- and late-Preg rats as well as non-Preg rat tissues treated with E2 + P4, and these effects were blocked by EMMPRIN antibody [48]. Also, endometrial expression of EMMPRIN and MMPs were regulated by ovarian sex hormones in cycling baboons and dysregulated in endometriotic baboons [491]. Additionally, EMMPRIN expression increases in the bovine endometrium during estrous cycle and early gestation [41]. Interestingly, whole blood analysis showed that EMMPRIN gene polymorphism rs424243T/G variant was overrepresented, while serum MMP-2 activity was reduced in PE compared to Norm-Preg women [69].

### 8.2. Effects of MMPs on Uterine Contraction Mechanisms

In human myometrial cells, oxytocin causes an initial [Ca^2+^]_c_ transient followed by low frequency [Ca^2+^]_c_ oscillations, which are attenuated by caffeine and the Ca^2+^ channel blockers verapamil and La^3+^ [492,493,494,495]. In rat uterine strips, oxytocin causes a steady contraction and superimposed phasic contractile responses [415,470,496]. Also, membrane depolarization stimulates Ca^2+^ influx and increases [Ca^2+^]_c_, causing uterine SMC contraction [497,498]. Oxytocin-induced [Ca^2+^]_c_ oscillations and phasic contractions in rat uterus were also reduced by the Ca^2+^ channel blocker nifedipine [496], suggesting a role of both Ca^2+^ release from the intracellular Ca^2+^ stores and voltage-dependent capacitative Ca^2+^ influx.

While MMPs largely promote ECM degradation and tissue remodeling, MMP inhibitors rapidly enhance contraction of Preg rat uterus, suggesting other biological effects. Also, MMP-2 and MMP-9 inhibit oxytocin-induced contraction in rat uterus [415], and α-adrenergic receptor-mediated contraction of rat blood vessels in the absence of detectable tissue degradation, likely through membrane hyperpolarization and reduction of Ca^2+^ influx [52,53,54]. Other studies showed reduced contraction and increased TWIK-related K^+^ channel (TREK-1) expression in human myometrium segments exposed to prolonged stretch, whereas P4 inhibited contraction and increased TREK-1 expression in late-Preg rat uterus [499,500]. Activation of BK_Ca_, SK_Ca_ and TREK-1 K^+^ channels and membrane hyperpolarization would reduce vascular and uterine contraction during Norm-Preg. In contrast, BK_Ca_ and SK_Ca_ channels are downregulated in placental arteries and HUVECs from PE patients, which may contribute to increased uteroplacental vascular resistance, RUPP, and FGR [501,502].

### 8.3. Dysregulation of Uterine MMPs and Preterm Birth

As fetal growth nears its completion in late pregnancy, uterine activity is first stabilized then increased in preparation for labor. MMP-1, MMP-2, MMP-3, MMP-7 and MMP-9 are found in the amniotic fluid and fetal membranes during Norm-Preg. MMP-2 and MMP-3 are expressed constitutively, while MMP-9 is barely detectable until labor. At labor, MMP-9 is the major gelatinase in fetal membranes, while MMP-2 is dominant in the decidua. Other studies showed upregulation of MMP-1, MMP-3, MMP-7, and MMP-8 during cervical ripening around the time of labor [503]. A disturbance in MMPs/TIMPs balance compounded by changes in sex hormone levels and their uterine receptors could increase uterine activity and lead to preterm birth [504].

Preterm birth complicates 10% to 15% of all pregnancies and is a leading cause of perinatal morbidity and mortality [15]. Risk factors for preterm birth include PE, hormonal imbalance, mental stress, malnutrition, antepartum Hemorrhage, uterine hyper-expansion in multiple pregnancy, and infection [16]. Other factors include polymorphism in angiogenic VEGF gene, with the risk of spontaneous preterm birth greater in women having the VEGF 936C/T or 936T/T allele than in women with VEGF 936C/C allele [505].

An increase in MMP-9 expression promotes ECM degradation in the fetal membranes and placenta, facilitating fetal membrane rupture and placental detachment [506]. Also, in women undergoing preterm cesarean delivery, samples of amniochorion and amniotic fluid showed increased expression/levels of MMP-2, MMP-3 MMP-9, and MT1-MMP and decreased expression of TIMP-2 in prematurely ruptured membranes compared with preterm labor membranes [15]. Other studies showed increased proMMP-2 and proMMP-9 levels in the amnion at term labor and even higher levels at preterm labor, supporting a role of MMP-2 and MMP-9 in regulating membrane rupture at term and preterm parturition [507]. Vaginal, cervical and intrauterine infection and microbial invasion of the amniotic cavity could also cause premature rupture of membranes and spontaneous preterm birth partly due to reduced levels of active MMP-2 [506,508]. In PE, decreased uterine active MMP-2 and MMP-9 could hinder uterine expansion and cause FGR and preterm birth [8,415]. Other studies showed that preterm parturition in the absence of microbial invasion of the amniotic cavity and intra-amniotic infection in both patients with preterm labor and patients with preterm premature rupture of membranes was associated with marked increases in MMP-7. MMP-7 is a physiologic constituent of amniotic fluid, and the increase in its levels with advancing gestational age and during microbial invasion of the amniotic cavity in preterm gestations may represent a maternal defense mechanism against infection and preterm birth [509]. Inflammation also induced myometrial AP-1 and increased the levels of stromelysins MMP-3 and MMP-10, resulting in preterm birth in mice [510]. Of note, plasma P4 levels were ~30% lower at 28 to 34 weeks gestation in women who delivered prematurely than in women who delivered at term [511], and progestin supplementation may prevent preterm birth [512]. The changes in MMPs in response to sex hormones and their potential role in dysregulation of uterine function and preterm birth need to be further examined.

## 9. Postpartum Effects of PE and Changes in MMPs

### 9.1. Maternal Changes, Postpartum Hemorrhage, Future Pregnancies, HTN

Following Norm-Preg and normal labor the body undergoes recovery in three stages: an initial period (6–12 h postpartum), subacute period (2–6 weeks after the initial period), and delayed postpartum period (over 6 months following the subacute period) [513]. In contrast, PE women may experience immediate, short-term and long-term postpartum complications.

PE may be associated with postpartum hemorrhage (PPH), which requires rapid replenishment of cryoprecipitate, a concentrated preparation of coagulation factors formulated from fresh frozen plasma [514]. Risk factors associated with severe PPH include IVF pregnancies, preexisting cardiovascular disease, PE, placental abruption, and uterine rupture [515]. Changes in MMPs and postpartum ECM remodeling could play a role in PPH and may represent a potential target to prevent this complication. Elevated antenatal plasma D-dimer levels have been detected in severe PE and are linked with PPH [516], likely through changing MMPs as they are positively correlated with serum MMP-9 levels in patients with acute type-A aortic dissection [517]. Also, analysis of urine proteins showed decreases in MMP-2 and MMP-9 levels 6–8 weeks postpartum compared to pre-delivery in PE but not Norm-Preg women [73].

PE effects could continue in a woman’s life and affect her next pregnancies. While typically the mother builds a greater maternal tolerance, a history of PE may be a risk for recurrent PE due to predisposing risk factors. In a study of Preg women who had a history of PE, 26.83% of the subjects developed recurrent PE in their second pregnancy, while 63.38–82.97% of subjects were normotensive in their subsequent pregnancies [518]. A separate study found that 71.3% out of 115 women with a history of PE had recurrent PE. Importantly, the women in their second pregnancy affected by PE experienced EO-PE and termination of pregnancy, higher BP, higher rate of proteinuria, and higher rate of complications compared to their first pregnancy. There was also a higher incidence rate of chronic HTN found in the recurrent PE group than the non-recurrence group, indicative of the long-term effects of PE. As for parturition, there was a higher rate of preterm birth and lower birth weights recorded from recurrent PE births [519]. It is possible that short-term alterations in the levels of bioactive circulating factors in women postpartum of a PE pregnancy can skew their basal set levels, increasing the chances of developing PE in future pregnancies. Interestingly, there was a 30% decrease in recurrent PE rates among women with a history of PE after the US Preventative Services Task Force recommended aspirin for PE prevention [520]. Aspirin inhibits prostaglandin synthase and reduces TXA_2_ levels but could also target coagulation factors and other mechanisms associated with the development of PE that need to be further examined.

The consequences of PE may persist long-term after a woman has passed her fertility window and even for the rest of her life. In a survey, 98% of women after experiencing PE reported that they were unaware of any long-term repercussions of PE and only 35% felt support in postpartum medical care [521]. By 1-year postpartum, the Preg woman is expected to achieve full recovery of cardiovascular-renal function and reversal of chamber diastolic dysfunction and impaired myocardial relaxation [522]. However, PE women may experience postpartum cardiovascular-renal complications. One study found that 63.4% of cesarean section cases of 142 patients with HTN-Preg and PE experienced immediate postpartum complications such as kidney infection [124]. In another study, postpartum PE women showed persistent left atrial enlargement, increased myocardial mass postpartum, and increased carotid intima-media thickness at ~12 years postpartum [523,524]. Another study showed that compared with Norm-Preg women, women with a history of EO-PE or LO-PE had enlarged aortic diameter 6 months to 4 years after delivery [525]. Also, in a study conducted within 7 days postpartum, nonsuperimposed PE women showed higher BP variability, and PE women with and without chronic HTN had lower heart rate variability compared to Norm-Preg counterparts, suggesting that the PE women cardiovascular system is under strain and has reduced capacity to respond to stress, placing them at a high risk for cardiac dysfunction [526]. Also, preterm PE women may have stage B asymptomatic heart failure postpartum that mentally and physically strain them further and place them at a high risk for cardiovascular events. Such complications could extend into and past the delayed postpartum period. Preterm PE subjects at 1 year postpartum may also exhibit asymptomatic left ventricular moderate to severe dysfunction and hypertrophy, which could be markedly higher in comparison to term PE and Norm-Preg women. There may also be a higher risk of developing HTN within 2 years postpartum in patients with preterm PE and subjects who show persistent left ventricular moderate to severe abnormal function and geometry [527]. Also, women with a history of preterm PE may show left ventricular systolic and diastolic function impairment observed at 1 year postpartum [527]. Microvascular reactivity measurements in the forearm using laser speckle contrast imaging, coupled with iontophoresis suggested that severe PE may be associated with heightened postpartum microvascular ACh-induced endothelium-dependent and SNP-induced endothelium-independent vasoreactivity and altered vascular function [528]. All factors affecting the mass and function of cardiovascular muscles and organs may likely contribute to a heightened premature risk for cardiovascular disease in women with a history of PE [529].

Studies on Preg rats showed an increase in perivascular, pericardial, and interstitial fibrosis in the heart that was reversed postpartum. Additionally, in late-Preg rats, MMP-1, MMP-2, and MMP-9 were downregulated and TIMP-1 and TIMP-4 were upregulated in the left ventricle. Postpartum expression of MMP-1, MMP-9, and TIMP-1 returned to non-Preg levels within 7 days, while MMP-2 levels remained lower, and TIMP-4 levels remained elevated postpartum [530]. Persistent changes in MMP-2 and TIMP-4 could have long-term effects on ECM remodeling after delivery. Preterm labor and cervical excision are both associated with PE, and studies in Preg mice showed that MMP-14 expression was increased postpartum in mice that experienced inflammation-induced preterm labor and cervical excision compared to mice that did not undergo preterm labor [531].

Persistent changes in circulating factors can alter the structure/function of cardiovascular organs. Elevated circulating levels of adhesion molecules ICAM-1 and VCAM-1 were detected in women with previous PE ~20 years postpartum, suggesting persistent inflammatory response and endothelial dysfunction [532]. Elevated plasma sFlt-1 levels were observed in women with previous PE at 5–8 years postpartum [533]. Also, serum sFlt-1 levels were greater in women with previous PE (~0.5 ng/mL) than previous Norm-Preg (~0.3 ng/mL), even more than 6 months following child birth [216]. Additionally, a positive correlation between circulating sFlt-1 and carotid intima-media thickness and left ventricular posterior wall thickness and a negative correlation between circulating PlGF and global longitudinal strain, carotid intima-media thickness, and mean arterial BP were found in women with previous PE at 12 years postpartum [534]. The levels of activin A, a member of the TGF-β superfamily, and the activin/follistatin-like 3 ratio were higher in women with PE than in women with uncomplicated pregnancies 10 years postpartum [535]. Activin A upregulates transcription factor SNAIL and MMP-2 expression in primary EVTs and could affect MMP-2 expression postpartum [536]. Other factors affecting MMP expression/activity postpartum should be further examined to determine their role in short-term and long-term maternal complications.

### 9.2. Neonatal Complications and in Utero Fetal Programming in PE

Neonates and the offspring from a pregnancy complicated by PE may also be affected by the condition. A study measuring neonatal outcomes from PE showed an 11.1% neonatal death rate accompanied with respiratory tract syndrome, asphyxia, and growth retardation [124]. One study suggested that women with HTN-Preg particularly those with severe PE or experiencing PPH may have an 11% higher likelihood of composite neonatal adverse outcome [537].

PE may also cause in utero programming that leaves changes in the offspring’s physiology and can have a permanent impact on the body metabolism and overall health [538]. Studies suggest that offspring of PE may be at a higher risk of developing cardiovascular, metabolic, and neurological diseases [539]. One study suggested that the risk of congenital heart defects could be greater in the offspring of PE than Norm-Preg, with a strong association for early and late preterm PE births [540]. This risk appeared to be physically manifested in offspring at 17.7 years after a PE birth, showing 0.025 greater cardiac relative wall thickness and 0.9 mL smaller left ventricular end-diastolic volume than their counterparts from Norm-Preg, indicative of an associated higher risk of coronary heart disease and stroke when those offspring reach adulthood [541]. However, larger scale studies are needed to confirm any relationship between PE and coronary heart disease risk in offspring [539]. One study showed that the offspring of PE had a 2.39-mmHg higher systolic and a 1.35-mmHg higher diastolic BP during childhood [542]. Another study suggested that offspring from PE could be at a higher risk of developing HTN compared to those from Norm-Preg [543].

Plasma testosterone levels are elevated in Preg women with PE and polycystic ovary syndrome (who often develop gestational HTN), and their offspring are at increased risk for HTN in adult life. Injection of testosterone to elevate its plasma levels 2-fold in gestational days 16 to 19 Preg rats increased BP, AT_1_R levels and the contractile response to Ang II in the mesenteric arteries [544]. Also, female rats prenatally exposed to elevated testosterone and examined at 6 month of age showed a decrease in aldosterone synthase (Cyp11b2) expression, reduced plasma aldosterone levels, and maintained plasma volume and Na^+^ and K^+^ balance, as well as increased BP and plasma levels of Arg^8^-vasopressin and Ang II, increased AT_1_R expression, decreased AT_2_R, and increased mesenteric arteries responsiveness to Ang II, suggesting potential compensatory mechanisms to maintain plasma volume and Na^+^ and K^+^ balance and mediate HTN in the adult female offspring [545].

Pathways leading to endothelial dysfunction and excessive vasoconstriction could also be impacted in the offspring of PE pregnancies. HUVECs from offspring of PE pregnancies show hypermethylation of IG-DMR, altered expression of imprinted genes *DLK1* and *MEG3*, decreased secretion of nitrites and EGF, and increased secretion of ET-1, indicative of endothelium dysfunction [546]. Also, C57BL/6J dams bred with E-V290M sires, which express a dominant-negative allele of PPARγ selectively in ECs, and infused with Arg-vasopressin throughout pregnancy showed elevated BP and urinary protein on gestational days 14–17. Systolic BP and ACh-induced relaxation were similar in offspring from vasopressin-infused and control pregnancies. However, male and female E-V290M offspring treated with a sub-pressor dose of Ang II showed impaired ACh-induced carotid artery relaxation that was improved by blocking ROCK signaling and incubation with a ROCK2-specific inhibitor, suggesting that endothelial PPARγ deficiency in offspring of HTN-Preg increases the risk for EC dysfunction when exposed to cardiovascular stressors in adulthood [547].

Whether vascular MMP expression/activity is altered and causes aberrant signaling and remodeling of blood vessels of offspring of PE pregnancies needs to be examined.

## 10. Prediction and Management of PE

LO-PE has a relatively long preclinical phase before clinically manifesting in late gestation, making it important to identify women at risk, proceed with early diagnosis using different biomarkers, and use interventional approaches to manage the disorder and improve the maternal and perinatal outcomes [11]. Decreased brachial artery flow-mediated vasodilation is an early indicator of EC dysfunction particularly between gestational weeks 24 and 28 and before the clinical diagnosis of PE. The predictive sensitivity of flow-mediated vasodilation is 87.5% for EO-PE and 95.5% for LO-PE [319]. Measurements of plasma levels of VEGF, PlGF, sFlt-1 and sEng can help in early detection of angiogenic imbalance in asymptomatic Preg women at high risk for PE [11]. Plasma sFlt-1 levels are elevated one month or longer before the onset of PE manifestations, and PlGF levels are decreased after the first trimester in Preg women who subsequently develop PE [548]. The sFlt-1/PlGF ratio is also higher in both EO-PE and LO-PE compared with Norm-Preg women [205] A meta-analysis of 20 different studies showed a relatively high overall diagnostic accuracy of sFlt-1/PlGF ratio for PE, with greater diagnostic efficiency in EO-PE than LO-PE [549].

Aberrant maternal immune response, increased monocytes and NK cells, altered cytokine levels, and elevated AT_1_-AA could be useful in predicting PE in early pregnancy [11]. Plasma TNF-α levels were elevated at gestational weeks 11–13 in women who later developed PE [550], with 100% sensitivity in predicting PE when combined with changes in uterine artery Doppler [551]. Plasma TNF-α levels could be more useful in predicting PE in the early third trimester than the first or second trimester [552]. While some studies showed that miRNA-206 was elevated in the plasma and placenta at gestational week 28 and could be involved in modulating several genes in women who develop PE [553], other studies question the predictive value of miRNAs [554]. Measurements of plasma levels of MMPs have not been consistent in PE, with some studies reporting elevated MMP-2 and MMP-9 [66] and other studies showing decreased MMP-9 levels [7], and measurements of localized MMPs in uteroplacental tissues and fluids may be more predictive of PE. Combined measurements of a cluster of biomarkers could carry a more predictive value of PE than a single biomarker.

Currently, there is no medical treatment for PE, and inducing labor is the most effective intervention. One antihypertensive agent such as methyldopa, a beta blocker (labetalol), or a Ca^2+^ channel blocker (nifedipine) can be used to alleviate HTN-Preg [555]. ACE inhibitors are not used due to their teratogenic effects, and atenolol and prazosin are not recommended. If PE progresses to eclampsia, magnesium sulfate is used to prevent seizures [556].

Targeting factors affecting EC dysfunction may promote vasodilation in HTN-Preg. Sildenafil may be useful in early-onset severe FGR, as it could promote fetal growth without causing major maternal adverse effects [557]. Sildenafil citrate enhances vasodilation in myometrial arteries isolated from Preg women with FGR, and restores EC integrity in placental vessels of L-NAME-treated HTN-Preg mice [558]. Eculizumab is an anti-C5 antibody that has been shown to stabilize the complement system, normalize bioactive factors and prolong pregnancy by 17 days in a woman with PE/HELLP syndrome, but could affect the immune response and increase susceptibility to infection [559]. In DOCA-salt HTN-Preg rats, pravastatin ameliorated HTN, increased placenta weight, reduced the levels of lipid peroxides and MMP-2, and augmented nitrates/nitrites levels and endothelium-dependent vasodilation [560]. Pravastatin also induced placental microsomal arginine uptake, causing rapid activation of eNOS and NO production, a mechanism that could reduce placental ischemia in HTN-Preg [561].

VEGF could ameliorate the angiogenic imbalance and vascular dysfunction in PE [193,562], but may increase vascular permeability and promote tumor angiogenesis [563]. Low molecular weight heparin therapy is associated with increases in circulating PlGF levels during the third trimester [564], and recombinant PlGF could be beneficial in PE [565]. Specific sFlt-1 antibodies could also reverse the angiogenic imbalance and improve the PlGF/sFlt-1 ratio in PE.

Counteracting the inflammatory response using TNF-α antagonists such as etanercept decreased BP, enhanced eNOS expression, reduced ET-1 levels and prevented cardiac changes in RUPP rats [247,566]. Also, IL-17 soluble receptor C reduced IL-17, prevented the recruitment of host defense cells, suppressed AT_1_-AA and ROS, reduced BP, and increased pup and placental weight in RUPP rats [311]. Infusion of anti-inflammatory IL-10 also decreased BP in DOCA/salt HTN-Preg rats [567].

Correcting the angiogenic imbalance and the inflammatory response could rectify the downstream aberrant vascular and uterine MMPs. Counteracting sFlt-1 by recombinant PlGF or sFlt-1 antibody should reverse the reduction in MMP-2 and MMP-9 and vascular signaling and remodeling in HTN-Preg. Interestingly, the anticoagulant protein tissue factor pathway inhibitor-2 (TFPI-2) is upregulated in PE serum and placenta, and in HTR-8/SVneo trophoblast cells exposed to hypoxia/reoxygenation. Downregulation of TFPI-2 increased trophoblast cell invasion and expression of MMP-2 and MMP-9, suggesting that TFPI-2 could be a target to improve trophoblast cell function through modulation of MMPs [568]. An alternative approach is to target MMPs. The MMP inhibitor doxycycline was thought to ameliorate HTN-Preg and vascular dysfunction in PE, but it decreased fetal and placenta weight and reduced trophoblast invasion and placental perfusion in HTN-Preg rats [261]. Novel approaches to correct MMP imbalance either directly or indirectly could be useful in the management of HTN-Preg and PE.

## 11. Conclusions

Norm-Preg is associated with uteroplacental and vascular remodeling in order to adapt for the growing fetus and the accompanying hemodynamic changes in the maternal circulation. Myometrium stretch and elevated sex hormones alter the expression/activity of specific MMPs, leading to extensive uteroplacental remodeling, uterine expansion, dilated spiral arteries and adequate blood flow to the fetus. Also, increases in vascular MMP-2 and MMP-9 promote vasodilation and decrease vascular resistance. However, genetic, immune, demographic and environmental risk factors could alter the maternal immune response, uteroplacental integrins, inflammatory cytokines and MMPs, causing inadequate placentation, shallow trophoblast invasion of spiral arteries, RUPP and placental ischemia/hypoxia. Animal models of HTN-Preg such as the RUPP rat have been useful in understanding the link between the localized placental ischemia and the generalized manifestations of PE. The ischemic/hypoxic placenta is believed to release the bioactive factors sFlt-1, sEng, TNF-α, IL-6, HIF, ROS and AT_1_-AA into the maternal circulation where they could target ECs causing endothelial dysfunction, decrease in the relaxing factors NO and EDHF, and increase in the constricting factor ET-1, or target VSMCs and enhances vasoconstriction, or ECM causing aberrant MMPs expression, collagen accumulation and arterial stiffness, leading to HTN-Preg. Also, localized release of bioactive factors in the uteroplacental tissues causes additional disruption of the MMPs/TIMPs balance, further restricting uteroplacental remodeling and blood flow, leading to FGR and preterm birth.

Measurements of maternal serum and placental levels of MMPs/TIMPs have not been consistent. Experimental studies showed specific changes in uterine, placental and aortic MMPs in HTN-Preg rats, but additional research should measure MMPs in other tissues particularly the small resistance vessels which affect BP. Also, the effects of angiogenic factors, cytokines, HIF, ROS and AT_1_-AA on MMPs/TIMPs ratio, MMP activity, ECM remodeling, arterial stiffness and vascular compliance through the course of pregnancy should be further examined.

All the currently available early diagnostic tools and measurements for management of PE are somewhat limited in their accuracy and efficiency. Further understanding of the interaction between bioactive factors, MMPs, vascular mediators and cellular mechanisms should help design more specific and efficient measures for prevention, early detection, and management of PE. Targeting a cluster of bioactive factors and MMPs rather than a single factor could be a more reliable approach for the detection and management of HTN-Preg, PE, and preterm birth. Importantly, targeting MMP imbalance as a potential therapeutic avenue in PE could pose a challenge as several MMPs could be involved and many MMP modulators lack specificity, making it critical to carefully examine any potential untoward off-target effects they may cause during pregnancy in large and thorough clinical trials. Also, research on how a PE pregnancy could affect the cardiovascular and metabolic health of the woman and her offspring long-term need to be further explored, and extended postpartum care should be provided.

## Figures and Tables

**Table 1:** MMP and TIMP levels in Norm-Preg and PE women.

Specimen (Units)	MMP TIMP	Norm-Preg	PE	EO-PE	LO-PE	Ref.
Plasma (ng/mL)	MMP-2	257 ± 123 ^SD^ 241.1 ± 35.3 ^SD^	290.5 ± 48.4 ^SD^	508 ± 137 ^SD^	491 ± 275 ^SD^	[65] [66]
	MMP-9	1391 ± 599 ^SD^ 240.0 ± 197.7 ^SD^	262.4 ± 153.8 ^SD^	1116 ± 389 ^SD^	834 ± 451 ^SD^	[65] [66]
	TIMP-1	142.8 ± 39.2 ^SD^	187.1 ± 35.4 ^SD^			[66]
	TIMP-2	158.3 ± 32.3 ^SD^	194.3 ± 49.3 ^SD^			[66]
Serum (ng/mL)	MMP-2	195.3 ± 43.5 ^SD^ 25.63 ± 4.56 ^SD^ 669 (560–760)	16.34 ± 7.07 ^SD^ 834 (656–1002)	242.9 ± 68.6 ^SD^	234.4 ± 79.2 ^SD^	[68] [69] [7]
	MMP-3	29.31 ± 58.79 ^SD^ 21.22 ± 23.28 ^SD^	52.81 ± 61.61 ^SD^	63.54 ± 71.58 ^SD^	27.91 ± 24.99 ^SD^	[68] [70]
	MMP-9	1752.5 ± 901.5 ^SD^ 390 (277–569)	290 (280–470)	1450.1 ± 838.0 ^SD^	1450.9 ± 1165.9 ^SD^	[68] [7]
	MMP-13	0.427 ± 0.228 ^SD^		0.590 ± 0.681 ^SD^	0.483 ± 0.278 ^SD^	[68]
	TIMP-1	148 (121–188)	213 (212–220)			[7]
	TIMP-2	228 (207–267)	232 (225–245)			[7]
Umbilical cord serum (pg/mL)	MMP-1	294.33 ± 11.53	124.68 ± 15.41			[71]
	TIMP-1	1304.20 ± 69.66	1363.00 ± 71.50			[71]
Placenta (mRNA)	MMP-2	7.6 ± 2.8 ^SD^	5.6 ± 1.5 ^SD^			[72]
	MMP-9	2.2 ± 2.6 ^SD^	−0.9 ± 2.0 ^SD^			[72]
Urine (ng/mg creatinine)	MMP-2	1.41 ± 0.22	3.03 ± 0.61			[73]
	MMP-9	0.09 ± 0.03	1.40 ± 0.39			[73]
	TIMP-2	2.10 ± 0.33	2.74 ± 0.70			[73]

EO-PE, early-onset PE, LO-PE, late-onset PE. Numbers represent means ± standard error of the mean, or means ± standard deviation ^SD^, or median (interquartile range) (minimum–maximum). Please note the discrepancies between studies such that each value should be compared with other values within the same study using the same assay, gestational age and PE subtype, at the same line with the corresponding reference.

**Table 2:** MMP levels in Norm-Preg versus RUPP rats.

Specimen (OD)	MMP	Norm-Preg	RUPP	Ref.
Uterus	MMP-1	~0.5	~0.8	[79]
	MMP-2	~1.0	~0.7	[8]
	MMP-7	~0.275	~0.55	[79]
	MMP-9	~0.4	~0.2	[8]
Placenta	MMP-1	~0.35	~0.75	[79]
	MMP-2	~0.9	~0.5	[8]
	MMP-7	~0.2	~0.35	[79]
	MMP-9	~0.3	~0.2	[8]
Aorta	MMP-1	~0.45	~0.78	[79]
	MMP-2	~0.9	~0.6	[8]
	MMP-7	~0.275	~0.55	[79]
	MMP-9	~0.4	~0.2	[8]

OD: optical densitometry of Western blot bands normalized to β-actin.

## Data Availability

No new data were created or analyzed in this study.

## Abbreviations

ACh	acetylcholine
Ang II	angiotensin II
AT_1_R	Ang II type 1 receptor
AT_1_-AA	Ang II AT_1_R agonistic autoantibodies
[Ca^2+^]_c_	cytosolic free Ca^2+^ concentration
cGMP	cyclic guanosine monophosphate
DOCA	deoxycorticosterone acetate
E2	estrogen, 17β-estradiol
ECM	extracellular matrix
EDHF	endothelium-derived hyperpolarizing factor
eNOS	endothelial nitric oxide synthase
EMMPRIN	extracellular MMP inducer
EGF	epidermal growth factor
ELISA	enzyme-linked immunosorbent assay
EO-PE	early-onset preeclampsia
ET-1	endothelin-1
EVT	Extravillous trophoblast
FGR	fetal growth restriction
H_2_O_2_	hydrogen peroxide
HIF	hypoxia-inducible factor
HELLP	hemolysis elevated liver enzymes low platelets
HTN-Preg	hypertension in pregnancy (hypertensive pregnant)
HUVECs	human umbilical vein endothelial cells
ICAM-1	intercellular adhesion molecule-1
IL-6	interleukin-6
IFN-γ	interferon-γ
KO	Knockout
L-NAME	N_ω_-nitro-L-arginine methyl ester
LO-PE	late-onset preeclampsia
MAPK	mitogen-activated protein kinase
MEGJ	myoendothelial gap junction
MMP	matrix metalloproteinase
Norm-Preg	normal pregnancy (normal pregnant)
NO	nitric oxide
O_2_^•−^	superoxide anion
P4	Progesterone
PE	preeclampsia
PGI_2_	prostacyclin
PlGF	placental growth factor
PKC	protein kinase C
PPH	postpartum hemorrhage
ROCK	Rho-kinase
ROS	reactive oxygen species
RUPP	reduced uterine perfusion pressure
sEng	soluble endoglin
sFlt-1	soluble fms-like tyrosine kinase-1
TGF-β	transforming growth factor-β
TIMP	tissue inhibitor of metalloproteinases
TNF-α	tumor necrosis factor-α
TXA_2_	thromboxane A_2_
VEGF	vascular endothelial growth factor
VCAM-1	vascular cell adhesion molecule-1
VSMC	vascular smooth muscle cell
WT	wild-type
